# Evidence of scaling advantage for the quantum approximate optimization algorithm on a classically intractable problem

**DOI:** 10.1126/sciadv.adm6761

**Published:** 2024-05-29

**Authors:** Ruslan Shaydulin, Changhao Li, Shouvanik Chakrabarti, Matthew DeCross, Dylan Herman, Niraj Kumar, Jeffrey Larson, Danylo Lykov, Pierre Minssen, Yue Sun, Yuri Alexeev, Joan M. Dreiling, John P. Gaebler, Thomas M. Gatterman, Justin A. Gerber, Kevin Gilmore, Dan Gresh, Nathan Hewitt, Chandler V. Horst, Shaohan Hu, Jacob Johansen, Mitchell Matheny, Tanner Mengle, Michael Mills, Steven A. Moses, Brian Neyenhuis, Peter Siegfried, Romina Yalovetzky, Marco Pistoia

**Affiliations:** ^1^ Global Technology Applied Research, JPMorgan Chase, New York, NY 10017, USA.; ^2^ Quantinuum, Broomfield, CO 80021, USA.; ^3^ Mathematics and Computer Science Division, Argonne National Laboratory, Lemont, IL 60439, USA.; ^4^ Computational Science Division, Argonne National Laboratory, Lemont, IL 60439, USA.

## Abstract

The quantum approximate optimization algorithm (QAOA) is a leading candidate algorithm for solving optimization problems on quantum computers. However, the potential of QAOA to tackle classically intractable problems remains unclear. Here, we perform an extensive numerical investigation of QAOA on the low autocorrelation binary sequences (LABS) problem, which is classically intractable even for moderately sized instances. We perform noiseless simulations with up to 40 qubits and observe that the runtime of QAOA with fixed parameters scales better than branch-and-bound solvers, which are the state-of-the-art exact solvers for LABS. The combination of QAOA with quantum minimum finding gives the best empirical scaling of any algorithm for the LABS problem. We demonstrate experimental progress in executing QAOA for the LABS problem using an algorithm-specific error detection scheme on Quantinuum trapped-ion processors. Our results provide evidence for the utility of QAOA as an algorithmic component that enables quantum speedups.

## Teaser

Exact simulation shows that the quantum approximate optimization algorithm provides a quantum speedup over best classical solvers.

## INTRODUCTION

Quantum computers have been shown to have the potential to speed up the solution of optimization problems. At the same time, only a small number of algorithmic primitives that provide broadly applicable speedups are known. These include amplitude amplification (*1*), quantum walks (*2*–*4*), and quantum Markov Chain algorithms (*5*, *6*), as well as the recently introduced short path algorithm (*7*, *8*).

The dearth of provable speedups in quantum optimization motivates the development of heuristics. A leading candidate for demonstrating a heuristic speedup in quantum optimization is the quantum approximate optimization algorithm (QAOA) (*9*, *10*). QAOA uses two operators applied in alternation *p* times to prepare a quantum state such that, upon measuring it, a high-quality solution to the problem is obtained with high probability. A pair of such operators is commonly referred to as one QAOA “layer.” The state is evolved with a diagonal Hamiltonian encoding the optimization problem by the first operator and with a nondiagonal transverse-field Hamiltonian by the second operator. Here, we consider the evolution times to be hyperparameters that are set by using a fixed, predetermined rule, analogously to the choice of a schedule in simulated annealing.

While QAOA has been studied extensively (*11*–*14*), little is known about its potential to provide a scaling advantage over classical solvers. A recent numerical study (*13*) of random 8-SAT with *N* ≤ 20 variables has shown that the time to solution (TTS) of QAOA with fixed parameters and constant depth grows as 1.23*^N^*. When QAOA is combined with amplitude amplification, the quantum TTS grows as 1.11*^N^* (*13*), whereas the best classical heuristic has TTS that grows as 1.25*^N^* (*13*).

Our work is motivated by this preliminary numerical evidence on small instances, which indicates that QAOA may potentially scale better than classical solvers when executed on an idealized quantum computer.

We study the scaling of QAOA TTS with the problem size on the low autocorrelation binary sequences (LABS) problem (*15*, *16*), also known as the Bernasconi model in statistical physics (*17*, *18*). The LABS problem has applications in communications engineering, where the low autocorrelation sequences are used for designing radar pulses (*15*, *19*). To solve LABS, one has to produce a sequence of *N* bits that minimizes a specific quartic objective.

We choose LABS to study the scaling of QAOA TTS for the following three reasons. First, the complexity of LABS grows rapidly, with optimal solutions known only for *N* ≤ 66 and the best heuristics producing approximate solutions of quality decaying with *N* for *N*200 (*20*, *21*). This makes it a promising candidate problem, since only a few hundred qubits are required to tackle classically intractable instances. Second, the performance of classical solvers for LABS has been benchmarked (*20*, *21*) in terms of the scaling of their TTS with problem size. Since optimal solutions are only known for *N* ≤ 66, the scaling of TTS for all classical solvers is obtained by fitting results for *N* ≤ 66. We reproduce these results and observe that that the scaling of classical solvers at *N* ≤ 40 matches the behavior for *N* up to 66 reported in the literature. This provides evidence that the scaling we observe for QAOA at *N* ≤ 40 will similarly extrapolate to larger *N*. Third, LABS has only one instance per problem size *N*. Combined with the hardness of LABS, this makes it possible to reliably study the scaling of QAOA at large problem sizes, where simulating tens or hundreds of random instances would be computationally infeasible.

We obtain the scaling by performing noiseless exact simulation of QAOA with fixed schedules. Our results are enabled by a custom algorithm-specific graphics processing unit (GPU) simulator (*22*), which we execute using up to 1024 GPUs per simulation on the Polaris supercomputer accessed through the Argonne Leadership Computing Facility. We find that the TTS of QAOA with number of layers *p* = 12 grows as 1.46*^N^*, which is improved to 1.21*^N^* if combined with quantum minimum finding. This scaling is better than that of the best classical heuristic, which has a TTS that grows as 1.34*^N^*. We note that we do not propose any new quantum algorithms in this work. Instead, we study a general quantum optimization heuristic with broad applicability (namely, QAOA) and make no specific modifications to adapt it to the LABS problem.

Our numerical evidence indicates that the proposed quantum algorithm scales better than the best classical heuristic in an idealized setting. However, we do not claim that QAOA is the best theoretically possible algorithm for the LABS problem. In particular, it may be possible to quadratically accelerate the best-known classical heuristic [Memetic Tabu (*23*)] by applying ideas similar to those used in quantum simulated annealing (*5*, *24*, *25*). Nonetheless, our results highlight the potential of QAOA to act as a useful algorithmic component that enables super-Grover quantum speedups.

As a first step toward execution of QAOA for the LABS problem, we implement QAOA on Quantinuum trapped-ion quantum processors (*26*, *27*) on problems with up to *N* = 18. We further implement an algorithm-specific error detection scheme inspired by Pauli error detection (*28*, *29*) and demonstrate that it can reduce the impact of noise on solution quality by up to 65%. Our experiments highlight the continuing improvements to quantum computing hardware and the potential of algorithm-specific techniques to reduce the overhead of error detection and correction.

## RESULTS

### Problem statement

We begin by formally defining the LABS problem, discussing the state of the art of classical LABS solvers, and describing how QAOA is applied to solve the problem.

For a given sequence of spins *s_i_* ∈ {±1}, the autocorrelation is given as

$$A_{k}\left(s\right)=\sum_{i=1}^{N-k}\;s_{i}s_{i+k}$$ (1)

The goal of the LABS problem is to find a sequence of spins that minimizes the so-called “sidelobe” energy

$${ℰ}_{\text{sidelobe}}\left(s\right)=\sum_{k=1}^{N-1}\;A_{k}^{2}\left(s\right)$$ (2)

or, equivalently, maximizes the merit factor

$$ℱ\left(s\right)=\frac{N^{2}}{2{ℰ}_{\text{sidelobe}}\left(s\right)}$$ (3)

**Fig. 1.**
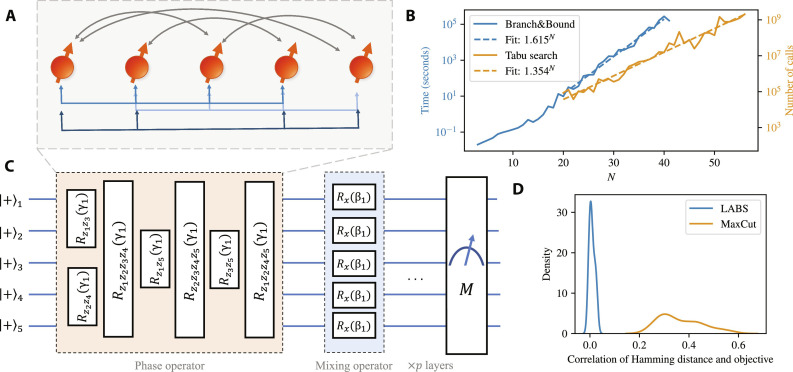
Classical and quantum algorithms applied to the LABS problem. (**A**) Diagram of the LABS problem (with example of *N* = 5). The problem involves nonlocal two-body (black lines) and four-body (blue lines) interactions. (**B**) Time to solution (TTS) of classical solvers. For the sizes considered, we observe clear exponential scaling with exponents matching their asymptotic values reported in the literature (see Table 1). (**C**) Diagram of QAOA circuit for a five-qubit example. Starting from a uniform superposition of the computational basis states, we apply *p* layers of phase and mixing operators, followed by measurement in the computational basis. (**D**) Distribution over 21 ≤ *N* ≤ 31 (for LABS) and 34 random instances (for MaxCut on random three-regular graphs with 20 nodes) of Pearson product-moment correlation coefficients relating the Hamming distance of bitstrings from the optimal solution with the objective value ofthe bitstring. LABS has a much lower correlation between the Hamming distance and objective, indicating that it is much harder than the commonly considered MaxCut problem.

The TTS is defined as the time a solver takes to produce this sequence. The energy ℰ_sidelobe_(**s**) is a polynomial containing terms of degrees 2 and 4, visualized in Fig. 1A. An instance of the LABS problem is unique for each *N* and can be encoded on qubits by the following Hamiltonian

$$H_{C}=2\sum_{i=1}^{N-3}\;z_{i}\;\sum_{t=1}^{\left\lfloor \frac{N-i-1}{2}\right\rfloor }\;\sum_{k=t+1}^{N-i-t}\;z_{i+t}z_{i+k}z_{i+k+t}+\sum_{i=1}^{N-2}\;z_{i}\sum_{k=1}^{\left\lfloor \frac{N-i}{2}\right\rfloor }\;z_{i+2k}$$ (4)

where *z_j_* is a Pauli *z* operator acting on qubit *j*.

The runtimes of state-of-the-art classical solvers for the LABS problem scale exponentially, with clear exponential scaling present at *N* ≤ 40 as shown in Fig. 1B. The best-known exact solvers are branch-and-bound methods that have a running time that scales as 1.73*^N^* (*21*). The best-known heuristic for general LABS is tabu search initialized with a memetic algorithm (Memetic Tabu) (*23*), and has a running time that scales as 1.34*^N^* (*30*). We provide a survey of classical solvers for the LABS problem in the Supplementary Materials.

To see why LABS is harder to solve than other commonly studied problems such as MaxCut, we can examine the correlation between the Hamming distance to the optimal solution and the objective. The comparison is shown in Fig. 1C. This correlation is one example of problem structure used by both classical and quantum heuristics to solve the problem quickly (*9*). The absence of this correlation highlights the hardness of LABS compared with other commonly considered problems such as MaxCut.

As a consequence of the exponential scaling, the LABS problem becomes classically intractable at moderate sizes. Specifically, the value of the best-known merit factor decreases notably for high *N*, whereas the asymptotic limit predicts that the merit factor should stay approximately constant. This failure of state-of-the-art heuristics has been observed for *N* > 200 (*20*, *21*). The clear failure of the classical method to obtain high-quality solutions even at small sizes makes LABS an appealing candidate problem for quantum optimization heuristics.

Here, we tackle the LABS problem using QAOA. As shown in the circuit diagram Fig. 1D, QAOA solves optimization problems by preparing a parameterized state

$$|\beta ,\gamma \rangle =\prod_{l=1}^{p}\;e^{-i\beta_{l}\sum_{j=1}^{N}\;x_{j}}e^{-i\gamma_{l}H_{C}}{|+\rangle }^{⊗N}$$ (5)

where ∣+〉^⊗*N*^ is a uniform superposition over computational basis states, *H_C_* is the diagonal Hamiltonian encoding the problem, and *x_j_* is a Pauli *x* operator acting on qubit *j*. The operator *e*^−*i*γ*H_C_*^ is commonly referred to as the phase operator, and $e^{-i\beta \sum_{j=1}^{N}\;x_{j}}$ as the mixing operator. The evolution times β, γ are hyperparameters chosen to maximize some figure of merit, such as the expected quality of the measurement outcomes or the probability of measuring the optimal solution. While β, γ can be optimized independently for each problem size, we consider them to be hyperparameters and use one fixed set of parameters for the LABS problem with a given QAOA depth *p* regardless of size. The fixed set of parameters is obtained by optimizing β, γ numerically for a number of small problem sizes and introducing an averaging and rescaling procedure to extrapolate parameters to any problem size (see Materials and Methods).

When choosing the parameters β, γ and evaluating the quality of the solution obtained by QAOA, two figures of merit are commonly considered. The first one is the expected merit factor of the sampled binary strings, given by

$${\langle C\rangle }_{\text{MF}}=\langle \beta ,\gamma |\frac{N^{2}}{2H_{C}}|\beta ,\gamma \rangle =\sum_{s\in {\left\{0,1\right\}}^{N}}\;\text{Pr}\left(s\right)ℱ\left(s\right)$$ (6)

We will refer to 〈*C*〉_MF_ as the “QAOA energy” as a shorthand. The second figure of merit is the probability of sampling the exact optimal solution, denoted by *p*^opt^ and equal to the sum of squared absolute values of amplitudes of basis states corresponding to exactly optimal solutions.

In the numerical experiments below, we follow the protocol of (*13*) and focus on scaling of the QAOA TTS with problem size *N* as the QAOA depth *p* is held constant. QAOA TTS is defined as $\frac{1}{p^{\text{opt}}}$ , i.e., the expected number of measurements required to obtain an optimal solution from the QAOA state. Boulebnane and Montanaro (*13*) rigorously show that, for random *k*-SAT, the runtime of constant-depth QAOA grows exponentially with *N* at any fixed *p*, with the scaling exponent depending on *p*. While the nature of the LABS problem makes it difficult to obtain analytical results analogous to (*13*), our numerical results also show clear exponential scaling of TTS. We note that, in practice, TTS of QAOA is $\Theta \left(N^{2}\right)\frac{1}{t}$ , where the Θ(*N*^2^) prefactor comes from the cost of implementing the LABS phase oracle (*31*). However, we do not include it in our analysis because it does not affect the scaling exponent.

### Scaling of quantum TTS for LABS problem

We now present the numerical results demonstrating the scaling of TTS of QAOA and QAOA augmented with quantum minimum finding (“QAOA+QMF”). The results are summarized in Table 1. Throughout this section, we present the numerical results obtained using exact noiseless simulations. The runtime scaling is obtained by evaluating QAOA once with fixed parameters β, γ (i.e., with no overhead of parameter optimization) and computing the value *p*^opt^ with high precision. We discuss the parameter setting procedure and the details of simulation in Materials and Methods.

**Table 1.** Scaling exponents for quantum and classical algorithms. Confidence intervals (CIs) are 95%. The reported asymptotic exponential scaling of classical state-of-the-art solvers is reproduced at *N* ≤ 40. For branch-and-bound, we include both the time to obtain a certificate of optimality [TTO reported in (*21*)] and the much shorter time to find an optimal solution (TTS). We observe that QAOA with constant depth of *p* = 12 augmented with quantum minimum finding (“QAOA+QMF”) has better TTS scaling than the best-known classical heuristics. Best scaling is indicated in boldface.

	QAOA+QMF	QAOA	Memetic Tabu		Branch-and-bound		
			Reproduced	(*23*, *30*)	Reproduced		TTO (*21*)
					TTS	TTO		
Fit	**1.21**	1.46	1.35	1.34	1.62	1.76	1.73
CI	(1.19, 1.23)	(1.42, 1.50)	(1.33, 1.38)	N/A	(1.57, 1.66)	(1.72, 1.79)	N/A

**Fig. 2.**
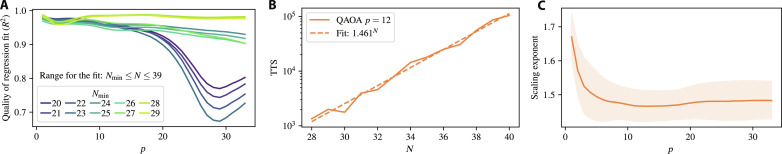
QAOA runtime scaling. (**A**) Quality of the exponential fit for different choices of minimum *N* to include in the fit. *N* ≥ 28 results in a robust fit, the quality of which does not deteriorate with *p*. *N* = 40 is omitted as it was only simulated up to *p* = 22. (**B**) TTS of QAOA at *p* = 12. Clear exponential scaling is observed. (**C**) Scaling exponent of QAOA runtime for different QAOA depths *p*. Shaded area shows 95% confidence interval. Increasing *p* beyond *p* ≈ 12 does not lead to better scaling.

We are interested in the scaling of the runtime of QAOA for large problem sizes *N*. An important question to address is the choice of the smallest *N* to include in the scaling analysis, since the algorithm’s behavior at small sizes may not be predictive of its behavior at large sizes. Note that the largest *N* we include is limited by the capability of the classical simulator. We use the quality of the fit as the criterion for the choice of the cutoff on *N*. Figure 2A shows that if we set the cutoff at *N* ≥ 28, we obtain a robust high-quality fit (*R*^2^ > 0.94), with the quality of the fit remaining stable as *p* grows. On the other hand, if smaller *N* values are included, the quality of fit begins to decay with *p*. Therefore, we include only *N* ≥ 28, obtaining the fit presented in Fig. 2B. We observe that TTS of QAOA grows as 1.46*^N^* with problem size at constant QAOA depth *p* = 12. We present evidence that the scaling exponent for QAOA at *p* = 12 is not sensitive to the choice of *N*_min_ in the Supplementary Materials.

As a quantum optimization heuristic with constant depth, on a fault-tolerant quantum computer, the QAOA performance can be improved by using amplitude amplification (*13*, *31*) or, more specifically, quantum minimum finding (*32*) (see Materials and Methods). The resulting scaling of TTS of QAOA augmented with quantum minimum finding (“QAOA+QMF”) is 1.21*^N^*.

**Fig. 3.**
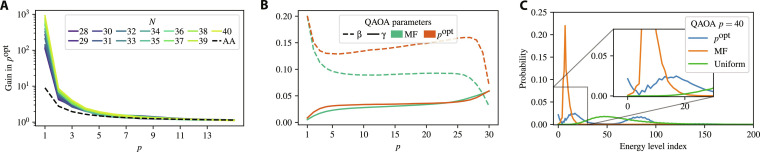
QAOA dynamics under different parameter schedules. (**A**) Gain in success probability *p*^opt^ from applying step *p* of QAOA and amplitude amplification (AA). The gain is defined as $p_{\text{at}\;\text{step}\;p}^{\text{opt}}/p_{\text{at}\;\text{step}\;\left(p-1\right)}^{\text{opt}}$ . The gain at *p* = 1 is over the random guess. Only one line is plotted for amplitude amplification since the lines for the values of *N* considered are visually indistinguishable. For small *p*, a QAOA layer gives orders of magnitude larger gain than a step of AA. (**B**) Fixed QAOA parameters for *p* = 30 chosen with respect to the QAOA energy 〈*C*〉_MF_ (“MF”) and probability of sampling the optimal solution (“*p*^opt^”). Different choice of optimization objective gives different resulting parameters. (**C**) Probability of obtaining a binary string corresponding to a given energy level of the LABS problem (the zeroth energy level is the ground state or optimal solution; lower is better). When parameters are optimized with respect to the expected merit factor (labeled “MF”), the QAOA output state is concentrated around the mean and fails to obtain a high overlap with the ground state. On the other hand, when parameters are optimized with respect to *p*^opt^ (labeled “*p*^opt^”), the QAOA state has a high overlap with both the ground state and higher energy states. The probability of obtaining the ground state is 27.3 times greater for QAOA with parameters optimized with respect to *p*^opt^ at *p* = 40.

We observe that, beyond a certain value (*p* ≈ 12), increasing QAOA depth does not lead to better scaling of TTS. This behavior is demonstrated in Fig. 2C. Consequently, running QAOA with *p* higher than 12 does not give any scaling advantage over amplitude amplification. This behavior is illustrated in Fig. 3A, which shows the increase in the success probability *p*^opt^ from applying a given step of QAOA and amplitude amplification. For amplitude amplification, at step *p*, we have $p^{\text{opt}}={\left\{\text{sin}\left[\left(2p+1\right)\text{arcsin}\sqrt{p_{0}}\right]\right\}}^{2}$ , where $p_{0}=\frac{8}{2^{N}}$ is the initial (random guess) success probability (*33*). Note that the 8 in the numerator is a consequence of a dihedral group symmetry, namely, *D*_4_. While asymptotically equivalent, amplitude amplification performs better than a realistic generalized minimum finding algorithm (*32*), as the formula used here considers the scenario where we know which states to amplify (i.e., the optimal merit factor is known). We observe that for small *p*, a step (layer) of QAOA gives orders of magnitude larger increase in success probability than does a step of amplitude amplification, implying an even larger improvement over direct application of quantum minimum finding. We provide additional details on comparison between QAOA and amplitude amplification in the Supplementary Materials.

We observe that the QAOA dynamics with parameters optimized for expected solution quality 〈*C*〉_MF_ and success probability *p*^opt^ are different. We plot the optimized parameters in Fig. 3B. We note that the parameters optimized with respect to one metric give performance that is far from optimal with respect to the other metric. This can be seen in Fig. 3C, which plots the energy distribution (with respect to the cost Hamiltonian) of the states appearing in the QAOA wave function weighted by probability. With the parameters optimized for ∣*C*〉_MF_, the QAOA output distribution is concentrated around its mean, and the overlap with the ground state or *p*^opt^ is very small. On the other hand, when the parameters are optimized with respect to *p*^opt^, the wave function is not concentrated and has large probability weight on the target ground state (i.e., high *p*^opt^). This comes at the cost of substantial overlap with high-energy states, which leads to poor expected solution quality. In the Supplementary Materials, we discuss the behavior of QAOA with parameters optimized with respect to different objectives.

### Experiments on trapped-ion system

We now present the experimental results demonstrating the algorithmic and hardware progress toward the practical implementation of QAOA. Implementation of the phase operator is especially challenging for currently available quantum processors. It requires a large number of geometrically nonlocal two-qubit gates, demanding high gate fidelity.

Recent progress in trapped-ion platforms based on the quantum charge-coupled device (QCCD) architecture (*26*, *27*) has led to a rapid increase in the number of qubits while maintaining high fidelity, enabling large-scale QAOA demonstrations (*34*, *35*), These systems implement two-qubit gates between arbitrary pairs of qubits by transporting ions into physically separate gate zones, resulting in high-fidelity two-qubit gates with low cross-talk. We leverage this progress to execute QAOA circuits for the LABS problem on Quantinuum H-series trapped-ion systems.

**Fig. 4.**
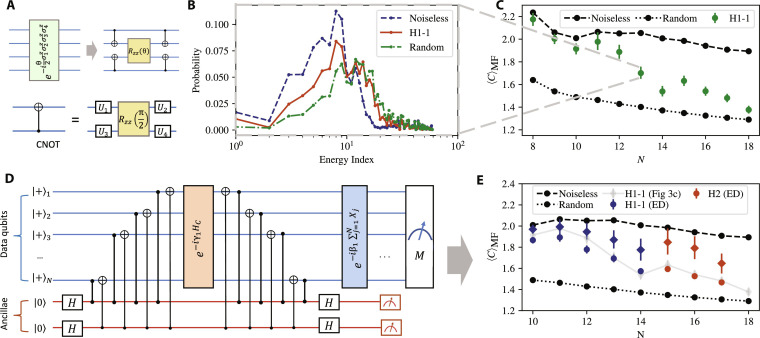
Experimental results on trapped-ion system. (**A**) Decomposition of four-body interaction terms into a two-body *R_zz_* gate and four two-body *cnot* gates, which can be realized via native *R_zz_* gates. (**B**) Energy density plot from experimental measured bitstrings for *N* = 13. Energy index is arranged in energy ascending order. As a comparison, the distributions for noiseless *p* = 1 QAOA simulation and random guess (assuming uniform distribution of all possible bitstrings) are shown. (**C**) Experimental results up to 18 qubits on a trapped-ion quantum device (H1-1) with QAOA layer *p* = 1 with optimized QAOA parameters. The error bars are calculated with 99% confidence intervals hereafter. (**D**) Illustration of parity check circuit. The *z* and *x* parities of states are mapped to ancillary qubits after implementation of full (or part of) phase operators via *cz* and *cnot* gates, respectively, followedby mid-circuit measurement on the ancillary qubits to extract the parity syndrome result. (**E**) Experimental results for circuit with parity check. Three mid-circuit *z*-parity and *x*-parity checks were performed using six ancillary qubits. The ancillae can also be reused after appropriate reset during the circuit. The red data points were run on the Quantinuum H2 hardware, while the blue data were from the H1-1 device. Data run on the H1-1 device without any ancillary qubits are shown in gray. Circles (diamonds) are the data without (with) postselection. The abbreviation ED refers to the error detection via the parity checks. Number of mid-circuit parity checks is fixed to be two for *N* = 10,11 and three for all other *N*. Improvement in expected merit factor after postselection according to parity syndrome measurement is observed.

To implement the QAOA circuit shown in Fig. 1D, we have to implement the phase operator. The four-body terms in the phase operator are decomposed into *cnot* gates and the native $R_{\mathrm{zz}}\left(\theta \right)=e^{-i\frac{\theta }{2}\mathrm{zz}}$ rotation as shown in Fig. 4A. To reduce the cost of implementing both the two-qubit and four-qubit interaction terms, we optimize the circuit by greedily canceling *cnot* gates (for algorithm details and gate count reduction, see the Supplementary Materials). The resulting circuit containing *cnot*s and *R_zz_*s is then transpiled into the two-qubit *R_zz_* gates and single-qubit gates that can be natively implemented by the trapped-ion system. We remark that the number of two-qubit gates is ≈10^3^ at *N* = 18, putting our experiments among the largest quantum optimization demonstrations on quantum hardware to date (*36*–*38*).

Here, we execute QAOA circuits with *p* = 1 using parameters β, γ optimized in noiseless simulation, followed by a projective measurement in the computational basis. In Fig. 4B, we show the energy probability distribution of measured bitstrings for *N* = 13. We observe a broad distribution due to the limited number of layers and experimental imperfections. Nevertheless, even at high *N*, where two-qubit gate count is high and the gate errors can be substantial, we observe a clear signal that indicates that QAOA is outperforming random guess. This is shown in Fig. 4C, which presents the experimentally obtained expected merit factors for various problem size up to *N* = 18. We note that the merit factor drops quickly for larger *N* and is approaching random guess because of experimental imperfections. We also note that at this scale LABS is easy for classical heuristics, which obtain optimal merit factors in <1 s. Implementing QAOA for LABS instances that are hard for classical solvers would likely require error correction, as the current implementation leads to an estimated two-qubit gate count of ≈7.5 × 10^5^ already at *N* = 67 and *p* = 12.

To improve the performance in the presence of noise, we implement an algorithm-specific error detection scheme. Since only the phase operator requires two-qubit gates, we focus on detecting errors that occur in the corresponding part of the circuit. Our scheme is based on the Pauli sandwiching error-detecting procedure of (*28*), which uses pairs of parity checks to detect some but not necessarily all errors that occur in a given part of the circuit. Following (*39*, *40*), we use the symmetries of the optimization problem to construct the parity checks. Specifically, we note that the LABS Hamiltonian preserves both *z* and *x* parities, that is, $\left[H_{C},{⊗}_{i}^{N}z_{i}\right]=\left[H_{C},{⊗}_{i}^{N}x_{i}\right]=0$ . We compute the parities onto ancillary qubits and perform mid-circuit measurement to determine whether an odd number of *z*- or *x*-flip errors occur during the circuit execution. The circuit with one check is shown in Fig. 4D. In the hardware experiments shown in Fig. 4E, we use up to three parity checks and observe consistent improvements in QAOA performance after postselecting on their outcomes. After postselection, the difference of merit factor between experimental results and noiseless simulation is reduced by 54% on average and up to 65% for specific *N*. In the Supplementary Materials, we present additional details on the error-detecting scheme performance, including how performance improves with the number of parity checks and the reduction in the algorithm runtime. We note that while error detection does not directly give samples with better merit factors, the potential improvement in runtime can be translated into performance gains at the algorithm level, for example, by being able to take more samples within a given time budget. In our experiments, in all but two cases, the optimal bitstring could be found within the postselected sample and, in all cases, within the total sample.

## DISCUSSION

Our main finding is that quantum minimum finding enhanced with QAOA scales better than the best-known classical heuristics for the LABS problem. This provides evidence for the potential of QAOA to act as a building block that provides algorithmic speedups on an idealized fault-tolerant quantum computer. We envision QAOA being used in a variety of algorithmic settings, similarly to how amplitude amplification acts as a subroutine in quantum algorithms for backtracking, branch-and-bound, and so on.

While our numerical evidence is only obtained by fitting instances with *N* ≤ 40, there are three observations that make us optimistic the observed scaling will hold for larger *N*. First, evaluating TTS is only possible when the solution is known, i.e., only up to 66 variables. This limitation applies equally to the classical solvers, which also report their scaling on *N* ≤ 66. Therefore, for the purposes of honest comparison between classical and quantum solvers, the relevant problem size is up to 66 variables, out of which our data cover up to 40 variables. Second, we note that for classical solvers considered, there is no change in scaling between *N* ≤ 40 and 40 ≤ *N* ≤ 66 (see Fig. 1B). This supports our claim that QAOA scaling will also remain the same up to at least *N* = 66. Finally, we note that there is a rich literature showing that QAOA performance at small finite *N* matches the rigorously derived infinite-size-limit behavior. This includes results for MaxCut (*41*), Sherrington-Kirkpatrick model (*12*), *k*-spin models (*42*), and random *k*-SAT (*13*). The results for *k*-SAT specifically focus on the scaling of TTS (*13*), matching our setting.

We take the first step toward the execution of QAOA for the LABS problem by implementing an algorithm-specific error detection scheme on a trapped-ion quantum processor. However, further improvements in quantum error correction and hardware are necessary to implement the quantum minimum finding augmented with QAOA. In particular, the overheads of fault tolerance (*43*) must be substantially reduced to realize the quantum speedup.

## MATERIALS AND METHODS

### Quantum minimum finding enhanced with QAOA

Here, we present the scaling results for QAOA combined with amplitude amplification (AA) or, more specifically, with quantum minimum finding (“QAOA+QMF” in Table 1). This reduces the scaling exponent by half as compared to directly sampling QAOA output. We now discuss in detail how QAOA is combined with the generalized quantum minimum finding algorithm of (*32*) to obtain the stated scaling.

We begin by noting that standard AA is not sufficient. This is because the LABS problem is framed as optimization and not search, i.e., there is no oracle for marking a global minimum. The trick for handling optimization is to perform a standard reduction from optimization to feasibility. The reduction is performed by introducing a threshold on the cost as a constraint and performing a binary search using AA as a subroutine. The oracle used by AA marks the elements below the current threshold. This reduction was first introduced by Dürr and Høyer (DH) (*1*). However, the quantum minimum finding algorithm of DH utilizes standard Grover search, i.e., it requires the initial state to be the uniform superposition. A modification to it is required to leverage the improved success probability afforded by QAOA.

Van Apeldoorn *et al*. (*32*) provided a simple extension of DH that allows arbitrary initial states, with the overall cost scaling inversely with overlap between the initial state and state encoding the optimal solution. We leverage this extension in our quantum algorithm. We use constant-depth QAOA to prepare the initial state for the quantum minimum finding algorithm. As QAOA state has overlap with the optimal state that is much larger than that of uniform superposition and scales more favorably, we obtain better performance than the direct minimum finding of DH. Specifically, we provide numerical evidence that our algorithm obtains a super-Grover speedup over exhaustive search for the LABS problem and scales better than the best-known classical heuristics. We present our modification to include QAOA for outputting an optimal solution *x*^∗^ to the LABS problem in Algorithm 1. It is based on the generalized minimum finding procedure outlined in lemma 48 of (*32*). To keep the current work self-contained, we include the analysis of the algorithm below. We will use the following standard quantum subroutine based on Grover search that searches for an element with unknown probability in a quantum state.

**Lemma 1.** [Exponential quantum search, (*44*)]. Let ∣ψ〉 = *U*∣0〉^⊗*N*^ be a quantum state in a 2*^N^*-dimensional Hilbert space with computational basis elements indexed by *N*-bit bitstrings, and *m* : {0,1}*^N^* → {0,1} be a marking function such that ∑_{*x*∣*m*(*x*)=1}_∣〈ψ∣*x*〉∣^2^ ≥ *p*. There exists a quantum algorithm **EQSearch**(*U*, *m*, δ) that outputs an element *x*^∗^ such that *m*(*x*^∗^) = 1 with probability at least δ using $O\left[\frac{1}{\sqrt{p}}\text{logleft}(\frac{1}{\delta })\right]$ applications of *U* and *m*.


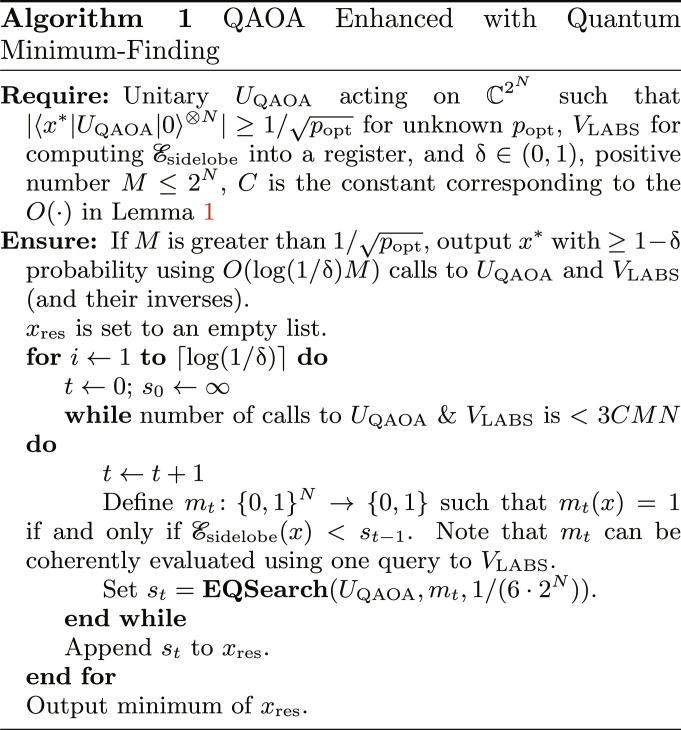


**Theorem 1.** Suppose a constant-depth QAOA circuit *U*_QAOA_ prepares a state ∣ψ〉 = *U*_QAOA_∣0〉^⊗*N*^ with *N* ≥ 3 such that we have $|\langle x^{*}|\psi \rangle |\geq 1/\sqrt{p_{\text{opt}}}$ , where ∣*x*^∗^〉 encodes an optimal solution to the *N*-bit LABS problem in a computational basis state, and we assume that *p*_opt_ ≥ 1/*N*. Then, running Algorithm 1 with parameters $M\geq 1/\sqrt{p_{\text{opt}}}$ and failure probability δ runs with a gate complexity of *O*(poly(*N*) log (1/δ)*M*) and finds *x*^∗^ with probability at least 1 − δ.

*Proof.* See the Supplementary Materials.

### Choice of QAOA parameters β, γ

Our strategy for setting the QAOA parameters β, γ used in our experiments is twofold. First, we optimize QAOA parameters for small *N* using the FOURIER reparameterization scheme of (*11*). Second, we use the optimized parameters for small *N* to compute fixed QAOA parameters that are then used for larger *N*. To apply the fixed parameters to an instance with a given size *N*, we rescale the parameters γ by *N*. We discuss the parameter optimization scheme and the parameter rescaling in the Supplementary Materials. We note that the results presented above can be improved if better parameter setting strategies are used.

**Fig. 5.**
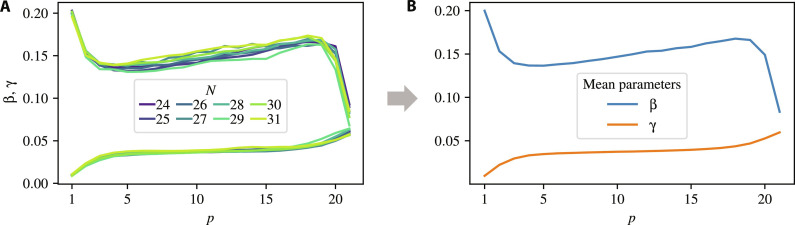
Visualization of how the fixed parameters are obtained. (**A**) Optimized QAOA parameters β (top lines) and γ (bottom lines) for *p* = 21. γ is multiplied by *N*/24 (constant factor of $\frac{1}{4}$ added for figure readability in both subfigures). (**B**) Fixed parameters obtained by taking the arithmetic mean over the optimized parameters.

The procedure for obtaining the set of fixed QAOA parameters is visualized in Fig. 5. Specifically, we optimize QAOA parameters for a set of small instances with sizes ${\left\{N_{j}\right\}}_{j=1}^{M}$ attainable in simulation and set the fixed parameters to be the mean over the optimized parameters

βFixed=1M∑j=1M βNj∗ (7)

γFixed=1M∑j=1M NjγNj∗ (8)

where $\beta_{N_{j}}^{*}$ , $\gamma_{N_{j}}^{*}$ are the QAOA parameters optimized for the LABS instance of size *N_j_* and *M* is the number of optimized instances. Then, the parameters used in QAOA for size *N* are given by $\beta^{\text{textFixed}},\frac{\gamma^{\text{Fixed}}}{N}$ . We use 24 ≤ *N_j_* ≤ 31 (*M* = 8).

### Error detection by symmetry verification

The error detection scheme relies on the symmetry of phase operator defined by Eq. 2. As it commutes with both ${⊗}_{i}^{N}z_{i}$ and ${⊗}_{i}^{N}x_{i}$ operators, one can measure the value of these operators and perform postselection on the measurement outcomes. That is, the state after the phase operator should have the same *z* and *x* parity as before it. In the presence of an odd number of bit flip or phase flip errors that occur during the implementation of phase operators, the resulting state will not be in the +1 eigenspace of the two syndrome operators.

Experimentally, we divide the whole phase operator into *m* splits such that each split has approximately the same number of two-qubit gates, and we perform syndrome checks at the end of each split to detect errors. The syndrome operators are mapped to ancillary qubits via sequential controlled-*x* or controlled-*z* gates and Hadamard gates applied before and after the partial phase operator. Since the number of two-qubit gates for the phase operators is higher than the number of gates used for the mapping by two to three orders of magnitude, additional errors introduced by ancillae are negligible. Furthermore, the cross-talk error probability during mid-circuit measurements is on the order of 10^−5^, considerably lower than the typical two-qubit gate infidelity of 2 × 10^−3^ for the trapped-ion systems we used (*27*). As a result, our error detection scheme leads to large improvements in QAOA performance on hardware at the cost of the number of repetitions growing exponentially with the number of checks (*28*). We note that the performance of the error detection scheme can be further improved by implementing parity checks using fault-tolerant constructions (*45*).

### Scaling of classical solvers

All scaling coefficients are obtained by fitting a least-squares linear regression on the logarithm of TTS. The confidence intervals on the scaling coefficients are obtained by using the Student’s *t* distribution and are reported with 95% confidence.

We use commercial state-of-the-art branch-and-bound solvers in numerical experiments. Figure 1B and Table 1 show results obtained using Gurobi (*46*), although we obtain similar results for CPLEX (*47*) (see the Supplementary Materials). The use of commercial branch-and-bound solvers is motivated by the observation that their scaling closely matches that reported in (*21*). Specifically, we observe that for both solvers the time to produce a certificate of optimality (TTO) scales with an exponent within a 95% confidence interval of the 1.73 exponent reported in (*21*). We note that unlike the solver presented in (*21*), commercial solvers are not parallelizable and can take advantage of only one central processing unit (CPU) with at most tens of cores. Since QAOA is a heuristic and does not guarantee optimality, we additionally run branch-and-bound solvers until a solution with an exactly optimal merit factor is found, at which point the execution is stopped. The resulting TTS scales more favorably: For Gurobi, the scaling is 1.615*^N^*, with a 95% confidence interval of (1.571,1.659). All the numbers reported correspond to the mean CPU time, with the mean taken over 100 random seeds for *N* ≤ 32 and 10 random seeds for *N* > 32. We present additional details of classical solver benchmarking in the Supplementary Materials.

Branch-and-bound algorithms are the best-known exact solvers for the LABS problem. In the regime where proving optimality is out of reach and the goal is simply to efficiently obtain sequences with high merit factors, heuristic algorithms are preferable. The best runtimes and runtime scaling reported in the literature (*23*) are from an algorithm known as Memetic Tabu. Memetic Tabu is a memetic algorithm, that is, an evolutionary algorithm augmented by local search. Specifically, an evolutionary algorithm is used to find initializations for tabu search, a metaheuristic that augments local neighborhood search with a data structure (known as the tabu list) that filters possible local moves if the potential solutions have been recently visited or diversification rules are violated (*48*). In terms of the runtime required to find optimal solutions in the regime where exact solutions have been found using branch-and-bound methods (*21*), Memetic Tabu has been observed to outperform both nonevolutionary methods and memetic algorithms that use simpler neighborhood search schemes such as steepest descent. To verify the scaling of tabu search on the regime of interest for comparison with QAOA, we use the implementation of Memetic Tabu in (*20*). For each length, we average the runtime over 50 random seeds, obtaining the scaling of the TTS of 1.35*^N^* with a 95% confidence interval of (1.33,1.38). This scaling closely matches the one reported in (*30*). We also note that solvers based on self-avoiding random walks (*20*) have been shown to be competitive with or outperform Memetic Tabu when the task is to find skew-symmetric sequences with the lowest autocorrelation. These solvers are specialized to search for skew-symmetric sequences and do not naturally extend to the unrestricted LABS problem.

### High-performance simulation of QAOA

Our numerical results are enabled by a custom scalable high-performance algorithm-specific QAOA simulator. We briefly describe the simulator here; for additional details and benchmarks comparing the developed simulator with the state-of-the-art methods for simulating QAOA, the reader is referred to (*22*).

Here, the main goal of the numerical simulation of QAOA is to evaluate the expectation of the cost Hamiltonian 〈*C*〉_MF_ and the success probability *p*^opt^. Since *p*^opt^ is exponentially small, it has to be evaluated with high precision. While many techniques can be leveraged for exact simulation, we opt to directly simulate the full quantum state as it is propagated through the QAOA circuit. We note in particular that tensor network techniques do not provide a benefit in this case since the circuit we simulate is deep and fully connected [see (*22*) for detailed comparison].

First, we leverage the observation that the cost Hamiltonian and hence the phase-separation operator are diagonal. This allows us to precompute the cost function evaluated at every binary input and multiply the exponentiated costs elementwise with the statevector to simulate the application of the phase-separation operator. This operation can be easily parallelized since it is an elementwise operation local to each element in the statevector. The same precomputed vector of cost function values is used to compute 〈*C*〉_MF_ by taking the inner product with the final QAOA state. The cost of precomputation is amortized over the large number of objective evaluations performed during parameter optimization and is thereby negligible.

Second, we note that the mixing operator consists of an application of a uniform *x* rotation applied on each qubit. Therefore, each rotation operation can be computed by multiplying a fixed 2 × 2 unitary matrix with a 2 × 2^*n*−1^ matrix constructed from reshaping the statevector. This step is parallelized by grouping the pairs of indices on which the 2 × 2 unitary is applied.

We perform the simulations on the Polaris supercomputer located in Argonne Leadership Computing Facility. We distribute the simulation to 256 Polaris nodes with four NVIDIA A100 GPUs on each node and one AMD EPYC CPU. The CPU is used to manage the communication and the assembly of final results. Each GPU hosts a chunk of the full statevector and a chunk of the integer cost operator vector. Application of the cost operator does not require any communication since it is local to each element. The grouping in the mixing operator depends on index *i* of the operator *x_i_* analogous to the grouping in the fast Walsh-Hadamard transform (*49*). For *i* ≤ *n* − log_2_(1024) = 29_,_ the pairing is local within each GPU. For *i* > 29_,_ we use CUDA-enabled MPI to distribute full chunks between nodes, which requires space to be reserved for two statevector chunks on each GPU.

## Supplementary Materials

### This PDF file includes:

Notes S1 to S4 Figs. S1 to S19 Table S1 References
