# Supplementary material for: Evidence of scaling advantage for the quantum approximate optimization algorithm on a classically intractable problem
Source: Sci Adv. 2024 May 29;10(22):eadm6761. doi: 10.1126/sciadv.adm6761 (PMC11135426; doi:10.1126/sciadv.adm6761)
Supplement: Supplementary file 1 — Notes S1 to S4 Figs. S1 to S19 Table S1 References [file sciadv.adm6761_sm.pdf]

Science Advances
AAAS

Supplementary Materials for

Evidence of scaling advantage for the quantum approximate optimization algorithm on a classically intractable problem

Ruslan Shaydulin *et al.*

Corresponding author: Ruslan Shaydulin, ruslan.shaydulin@jpmchase.com

Sci. Adv. **10**, eadm6761 (2024)
DOI: 10.1126/sciadv.adm6761

This PDF file includes:

Notes S1 to S4
Figs. S1 to S19
Table S1
References

S1. BACKGROUND ON THE LABS PROBLEM

The problem of finding sequences with low sidelobe energies attracted attention in the 1960s and 1970s due to its applications to the reduction of the peak power of radar pulses (15, 16). The merit factor F was first introduced by Golay (50) and defined as the ratio of central to sidelobe energy of a binary sequence. Improved merit factors were obtained over the years by exhaustive (19, 51) and non-exhaustive (52) search methods. Explicit sequences asymptotically achieving the merit factor of $\mathcal{F} \approx 6.34$ are known (53). The conjectured asymptotic limit of $\mathcal{F} \to 12.32$ as $N \to \infty$ was derived using arguments from statistical mechanics in Ref. (54). Bernasconi reframed the LABS problem as a spin model with long-range 4-spin interactions to apply simulated annealing to it (17). However, simulated annealing failed to obtain high-quality solutions, with the failure attributed to the "golf-course type" energy landscape (17). Bernasconi further conjectured that this property of the landscape will prevent stochastic search procedures from obtaining high-quality solutions for long sequences (17). While the presence of isolated deep and narrow ("golf-course type") global minima has been debated (55), the conjecture that stochastic search procedures will fail has held up so far.

A commonly considered class of sequences are those exhibiting skew-symmetry, which for sequences of odd length $N = 2k-1$ are defined as $s_{k+l} = (-1)^l s_{k-l},\ l \in \{1, \ldots, k-1\}$. Skew-symmetric sequences are known to be optimal for many odd N instances. Since skew-symmetry reduces the search space from 2^N to $2^{\frac{N}{2}}$, only searching this subspace leads to better runtime scaling. Therefore many algorithms are restricted to only searching this subspace. The best-known heuristic for skew-symmetric LABS uses a sequence of self-avoiding walk segments when searching and has a running time that scales as 1.15^N.(30) In this work, we target the general LABS problem and therefore we do not consider solvers that are only capable of tackling the skew-symmetric instances.

To identify the best classical solvers for the LABS problem, we have performed an extensive literature review. Table S1 summarizes this review. We also note recent works targeting a peak sidelobe value rather than merit factor (56–58). The ideas from these works may be fruitfully applied to the LABS problem in the future.

Year	Ref.	Kind	General	Scaling	$N_{\max}$	Algorithm
1975	(59)	Exact	Yes	N/A	40	Exhaustive search
1977	(19)	Exact	No	N/A	59	Exhaustive search
1985	(52)	Heuristic	No	N/A	199	Non-exhaustive search
1990	(51)	Exact	No	N/A	69	Exhaustive search
1990	(51)	Heuristic	No	N/A	117	Non-exhaustive search
1996	(60)	Exact	Yes	1.85^N	48	Branch-and-bound
2003	(61)	Heuristic	Yes	1.463^N	64	Kernighan-Lin
2003	(61)	Heuristic	Yes	1.397^N	47	Evolutionary
2006	(62)	Heuristic	Yes	N/A	48	Tabu search
2009	(23)	Heuristic	No	1.17^N (30)	201	Memetic Tabu
**2009**	**(23)**	**Heuristic**	**Yes**	$\mathbf{1.34^N}$ (30)	85	**Memetic Tabu**
2012	(63)	Exact	No	N/A	30	Mixed-integer quadratic programming
2012	(63)	Exact	Yes	N/A	51	Mixed-integer quadratic programming
2013	(64)	Exact	No	1.337^N (30)	89	Branch-and-bound
**2016**	**(21)**	**Exact**	**Yes**	$\mathbf{1.73^N}$	66	**Branch-and-bound**
2017	(30)	Heuristic	No	1.15^N	400	Self-avoiding walks
2018	(65)	Heuristic	No	1.18^N	225	Stochastic search

TABLE S1. Select prior works tackling the LABS problem with classical solvers. Best results to date for general LABS are highlighted in bold. "General" column signifies whether a solver tackles general LABS or only skew-symmetric ones. The latter are only included for reference as we consider general LABS in this work. $N_{\max}$ is the largest size tackled (but not necessarly solved exactly). We note that in Ref. (61), Kernighan-Lin solver has worse scaling but better constants, which is why it was used for larger N experiments. We do not include in this table negative results, such as results showing that simulated annealing (17, 55) and plain evolutionary algorithms (66) fail to obtain good solutions.

S2. QAOA AS AN EXACT AND APPROXIMATE OPTIMIZATION ALGORITHM

We now provide additional numerical results highlighting the differences in QAOA behavior with parameters optimized for approximate and exact solutions. In this work, we use QAOA as an exact solver, with time to solution as the target metric. However, QAOA is typically used as an approximation algorithm, (10) with most theoretical results focusing on the expected solution quality obtained by QAOA. For example, this is the setting used in recent results ruling out quantum advantage in approximation with log-depth local quantum algorithms like QAOA (67–69). These results do not apply to our chosen setting of using QAOA as an exact solver. In fact, on the LABS problem, we observe that QAOA can provide poor approximations in polynomial time while still offering speedups as an exact exponential-time solver.

We begin by discussing the parameters themselves. QAOA parameters are typically chosen with respect to QAOA energy (10, 11, 70–75) or the probability of sampling the optimal solution (13). Figure S1a shows the difference in QAOA parameters in the two scenarios for the specific case of the LABS problem. First, we note that QAOA parameters that maximize $\langle C\rangle_{\mathrm{MF}}$ are substantially different from those maximizing p^{opt}. Specifically, while the values of $\boldsymbol{\gamma}$ are similar, the value of $\boldsymbol{\beta}$ for p^{opt} is much larger than that for $\langle C\rangle_{\mathrm{MF}}$. Qualitatively, this implies that the probability amplitudes are allowed to "mix" more, making QAOA state not concentrated with respect to Hamming distance. This behavior is seen when examining the QAOA output distribution, which is shown for both parameter schedules in Fig. S2 and discussed in detail below.

Second, QAOA parameters that give good performance with respect to one metric are far from optimal with respect to the other metric. Fig. S1b shows QAOA performance with parameters linearly extrapolated between the parameters $(\boldsymbol{\beta}^{\langle C\rangle_{\mathrm{MF}}}, \boldsymbol{\gamma}^{\langle C\rangle_{\mathrm{MF}}})$ that give a high value of $\langle C\rangle_{\mathrm{MF}}$ and $(\boldsymbol{\beta}^{p^{\mathrm{opt}}}, \boldsymbol{\gamma}^{p^{\mathrm{opt}}})$ that give high p^{opt} : $\boldsymbol{\gamma} = t\boldsymbol{\gamma}^{p^{\mathrm{opt}}} + (1-t)\boldsymbol{\gamma}^{\langle C\rangle_{\mathrm{MF}}}$ and $\boldsymbol{\beta} = t\boldsymbol{\beta}^{p^{\mathrm{opt}}} + (1-t)\boldsymbol{\beta}^{\langle C\rangle_{\mathrm{MF}}}$. We note that the parameters $\boldsymbol{\beta}^{\langle C\rangle_{\mathrm{MF}}}, \boldsymbol{\gamma}^{\langle C\rangle_{\mathrm{MF}}}$ $(t=1)$ give a very low value of p^{opt} and vice versa. This suggests that substantial performance gains are possible if parameters are chosen differently with respect to the two figures of merit, rather than using one as a proxy for the other as is commonly done in QAOA research.(76, 77) Similar observations have been made in Refs. (78 and 79), though the difference observed between the two figures of merit is more drastic in our case due to the hardness of the problem considered.

In the numerical experiments presented in the main test, we use time to solution as the target metric. For completeness, we include the results showing QAOA performance as an approximation algorithm. We observe that QAOA performs poorly on the LABS problem with respect to the expected merit factor $\langle C\rangle_{\mathrm{MF}}$. Specifically, we observe that QAOA fails to outperform even simple classical techniques at high depth. Fig. S3 shows the expected merit factor of QAOA for fixed $p = 100$ as a function of N, as well as examples of how the expected merit factor grows with p for two fixed values of N. We observe that as N grows, QAOA at $p = 100$ achieves an expected merit factor $\langle C\rangle_{\mathrm{MF}}$ of less than 5. Note that explicit analytical sequences achieving merit factor > 6 are known (53). Moreover, we see that $\langle C\rangle_{\mathrm{MF}}$ grows increasingly slowly as N and p increase, suggesting that a prohibitively high value of p would be required to achieve a high expected merit factor.

We can understand this behavior by examining the values of the merit factor attainable by binary strings (in physics

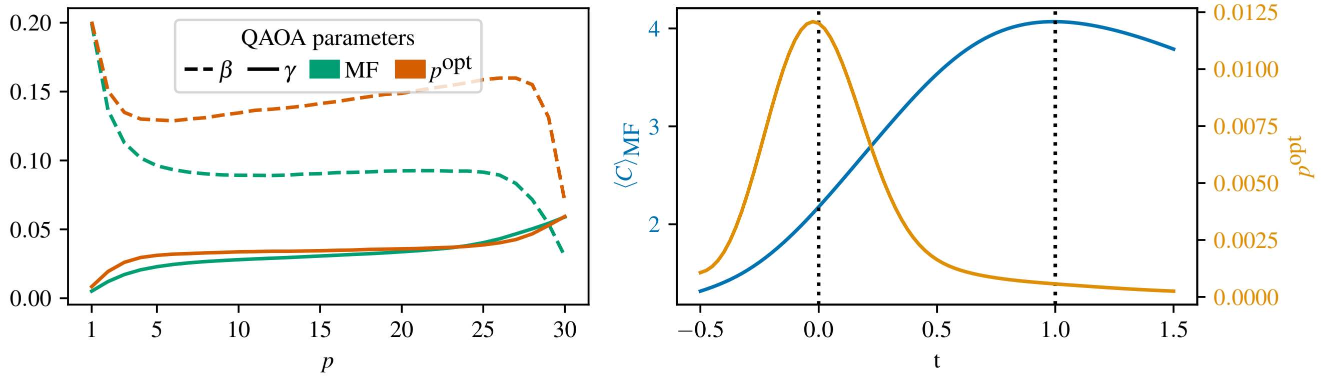

FIG. S1. **QAOA parameters optimized for different objectives a,** Fixed QAOA parameters for $p = 30$ chosen with respect to the QAOA energy $\langle C\rangle_{\mathrm{MF}}$ ("MF") and probability of sampling the optimal solution ("p^{opt}"). When the parameters are optimized with respect to p^{opt}, the value of $\boldsymbol{\beta}$ is substantially larger throughout QAOA evolution. Subfigure reproduced from the main text. **b,** QAOA performance for $N = 25$, $p = 30$ with parameters linearly extrapolated between fixed parameters for p^{opt} $(t = 0)$ and $\langle C\rangle_{\mathrm{MF}}$ $(t = 1)$. QAOA parameters optimized for $\langle C\rangle_{\mathrm{MF}}$ give very poor values of p^{opt} and vice versa.

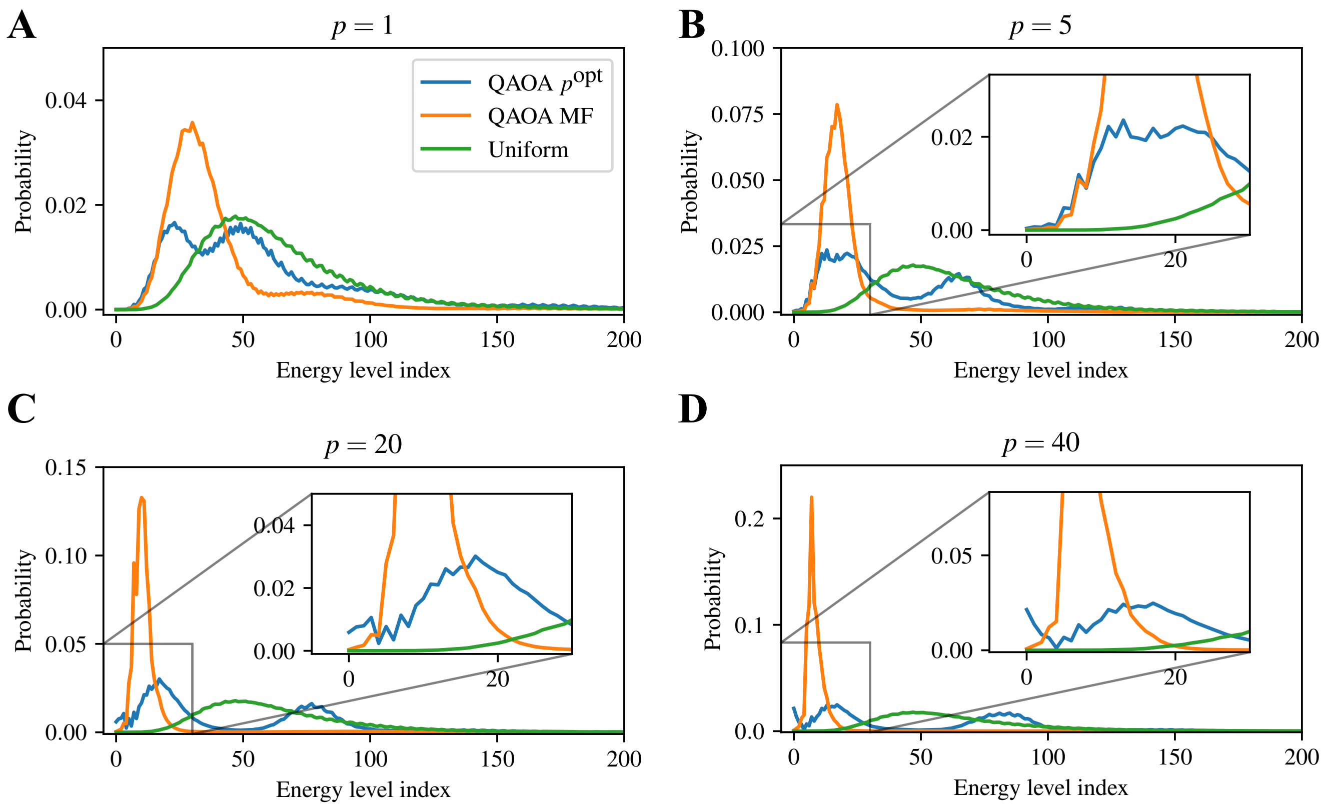

FIG. S2. **QAOA dynamics with parameters optimized for different objectives** The probability of obtaining a binary string corresponding to a given energy level of the LABS problem (the zeroth energy level is the ground state or optimal solution, lower is better) for varying p (**A-D**). When parameters are optimized with respect to the expected merit factor (labeled "QAOA MF"), the QAOA output state is concentrated around the mean and fails to obtain a high overlap with the ground state. On the other hand, when parameters are optimized with respect to p^{opt} (labeled "QAOA p^{opt}"), the QAOA state has a high overlap with both ground state and higher energy states. The probability of obtaining the ground state is 27.3 times greater for QAOA with parameters optimized with respect to p^{opt} at $p = 40$ (**D**).

terms, we are examining the spectrum of the LABS Hamiltonian). For the $N = 25$ problem, presented in Fig. S2, only the 4 lowest energy levels correspond to a merit factor better than 6. This means that QAOA must concentrate all the wave function mass on a superposition of a small number of computational basis states. Due to QAOA preserving the D_4 symmetry of the problem (80), this is a highly entangled state, which is hard to prepare (81). As a result, QAOA requires a very large value of p to obtain a high expected merit factor. If, on the other hand, we choose the probability of sampling the exact optimal solution p^{opt} (overlap between QAOA state and the ground state of the LABS Hamiltonian) as the target metric, a much lower value of p is needed to obtain good success probability. Qualitatively, Fig. S2 shows how this state preparation succeeds by allowing a substantial part of the QAOA state to "leak" to high energy levels. This can be observed by noticing how the population of energy levels > 50 stays relatively high for QAOA with parameters optimized with respect to p^{opt}, but becomes negligible if QAOA parameters are chosen with respect to $\langle C \rangle_{\mathrm{MF}}$.

The success of QAOA in preparing states with a large overlap with the ground state of the LABS Hamiltonian (i.e. states having a high probability of measuring exactly optimal solution) motivates the choice of time to solution as the target metric for QAOA evaluation. We show the time to solution at the largest p explored numerically ($p = 33$) in Figure S4. We remark that the QAOA succeeds at achieving high overlap at $N \leq 40$.

Our results suggest a new way of viewing the potential of QAOA to provide algorithmic speedups and provide an important caveat to theoretical results bounding the approximations attainable by QAOA with constant depth.(42) Even in the regime where the expected solution quality of QAOA is bounded, it can be still useful as a tool to obtain a high probability of measuring the exact optimal solution.

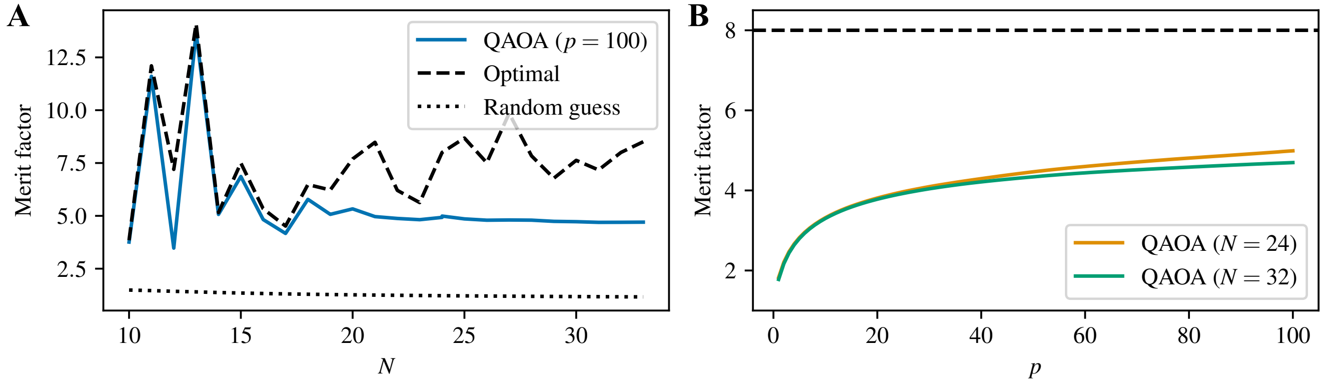

FIG. S3. **QAOA performs poorly as an approximation algorithm A,** Performance of QAOA as an approximation algorithm. Explicit constructions of skew-symmetric sequences exist that achieve $\mathcal{F} \approx 6.34$ for large N.(53) Simulated annealing achieves $\mathcal{F} \approx 5$ for large N.(17) For QAOA, the expected value of merit factor $\langle C \rangle_{\mathrm{MF}}$ is plotted. The expected merit factor of QAOA output is below the values easily attainable classically. **B,** For both $N = 24$ and $N = 32$, the optimal merit factor is 8 (dashed line).

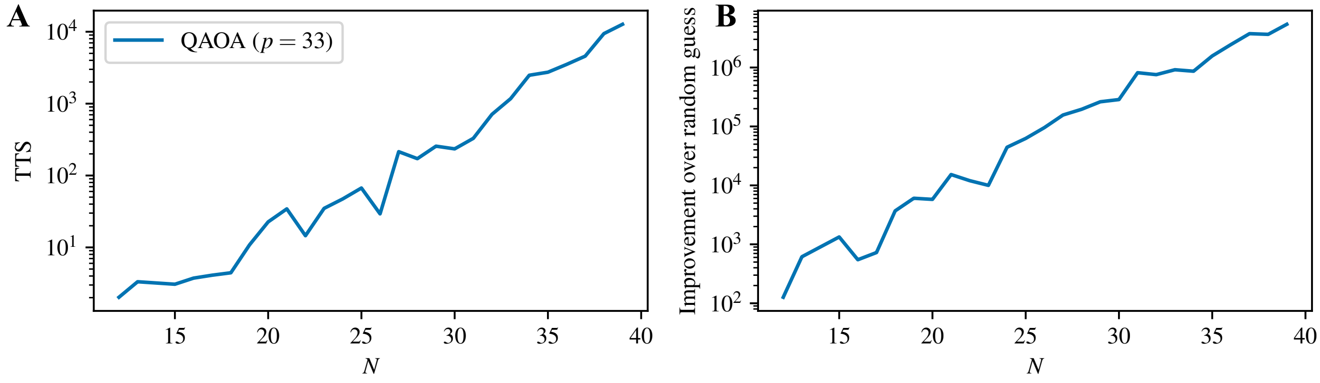

FIG. S4. **QAOA achieves high overlap A,** Time-to-solution (TTS) of QAOA with parameters chosen with respect to p^{opt}. **B,** Improvement over random guess. For the largest case numerically considered, which is the $N = 39$ problem at $p = 33$, the expected number of shots required to solve it is 1.2×10^4. This is a 5.4×10^6 factor improvement over random guess.

S3. DETAILS OF NUMERICAL STUDIES

We now present in detail how the fixed parameters were chosen. Our procedure for doing so is as follows. First, we optimize the QAOA parameters using the FOURIER (11) reparameterization. We show evidence that the optimization of the reparameterized QAOA gives the same performance as the more extensive optimization of the standard parameterization. Second, we set our fixed parameters to be the arithmetic mean of the (appropriately rescaled) optimized parameters for smaller N. We provide evidence that for smaller N where directly optimized parameters are available, the fixed parameters lead to QAOA performance that is close to that with the optimized parameters. We note that better parameter setting schemes can only improve our performance.

A. Optimized QAOA parameters for LABS change with N

First, we observe that the optimized QAOA parameters are not invariant with N. Specifically, we observe that the optimized value of $\boldsymbol{\gamma}^*$ goes down with N as $\frac{1}{N}$. S5A plots this for $p = 1$. We note that $\boldsymbol{\beta}^*$ is roughly constant with N. We observe this scaling for all p. As an example, S5B plots optimized parameters for two different values of N. After rescaling $\boldsymbol{\gamma}^*$ by N, the two sets of parameters are visually indistinguishable. Below, we take advantage of this scaling of $\boldsymbol{\gamma}^*$ in two ways. First, we improve the convergence of local optimization runs by rescaling the initialization

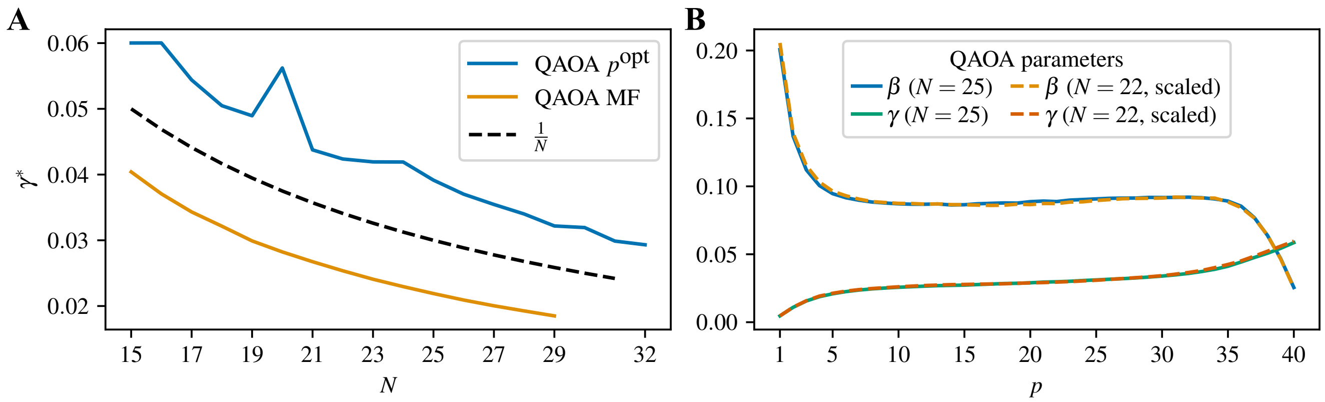

FIG. S5. **Scaling of QAOA parameters with problem size A,** Scaling of the optimized value of QAOA parameter γ with N for $p = 1$ when optimized with respect to expected merit factor $\langle C \rangle_{\text{MF}}$ ("QAOA MF") and probability of obtaining optimal solution p^{opt} ("QAOA p^{opt}"). Optimized γ^* decreases with N as $\frac{1}{N}$. **B,** QAOA parameters optimized with respect to $\langle C \rangle_{\text{MF}}$ for $N = \in \{22, 25\}$. For $N = 22$, the parameters $\boldsymbol{\gamma}$ are scaled by 22/25. After rescaling, the parameters for $N = 22$ and $N = 25$ are visually indistinguishable.

and the initial step size of the local optimizer. Second, we use it to correctly account for scale when executing QAOA with fixed parameters.

The scaling of $\boldsymbol{\gamma}^*$ with extremal properties of the objective function has been observed before for other problems. For example, the normalized value of the maximum cut on D-regular graphs grows with D (to the first order) and $\boldsymbol{\gamma}^*$ decreases as $\frac{1}{\sqrt{D}}$.(12, 82) Analogous scaling has been observed for weighted problems.(14) In LABS, the energy $\mathcal{E}_{\text{sidelobe}}(\boldsymbol{s})$ grows as N^2, so $\boldsymbol{\gamma}^*$ decreases as $\frac{1}{N}$. Formally establishing this connection is a promising direction for future research.

B. QAOA parameter optimization with FOURIER scheme

For a given figure of merit, we optimize the QAOA parameters as follows. In all cases below, we use the nlopt (83) implementation of BOBYQA (84) gradient-free local optimization algorithm. In all cases, we run BOBYQA until convergence, with convergence specified by relative tolerances on changes in parameters and in objective function value of 10^{-8}. BOBYQA has been shown to outperform other local optimizers on the task of optimizing QAOA parameters (70). We expect similar results with any other local hill-climbing algorithm, albeit at a potentially different cost in terms of the number of iterations.

For $p = 1$, we optimize the QAOA parameter exhaustively by running the local optimizer from 400 initial points. We set the initial step size (`rhobeg`) to $0.01/N$. The initial points $\beta^{\text{init}}, \gamma^{\text{init}}$ are chosen uniformly at random from $\beta^{\text{init}} \in [0.1, 0.2], \gamma^{\text{init}} \in [0, 0.85/N]$ when optimizing with respect to $\langle C \rangle_{\text{MF}}$, and $\beta^{\text{init}} \in [0.15, 0.3], \gamma^{\text{init}} \in [0.6, 1.2/N]$ when optimizing with respect to p^{opt}. The regions for initializations are read off from grid search results for $p = 1$.

For $p > 1$, we follow the FOURIER$[\infty, 0]$ scheme of Ref. (11). Specifically, we change the QAOA parameterization to the frequency domain as follows:

$$\gamma_i = \sum_{k=1}^{p} u_k \sin\left[\left(k - \frac{1}{2}\right)\left(i - \frac{1}{2}\right)\frac{\pi}{p}\right], \tag{S1}$$

$$\beta_i = \sum_{k=1}^{p} v_k \cos\left[\left(k - \frac{1}{2}\right)\left(i - \frac{1}{2}\right)\frac{\pi}{p}\right]. \tag{S2}$$

Then we optimize over the new parameters $\boldsymbol{u}, \boldsymbol{v}$. We take the optimized parameters $\boldsymbol{u}^*_{p-1}, \boldsymbol{v}^*_{p-1}$ for $p - 1$ and run one local optimization from $\boldsymbol{u}^*_p = (u^*_{p-1}, 0)$, $\boldsymbol{v}^*_p = (\boldsymbol{v}^*_{p-1}, 0)$. The initial step size (`rhobeg`) for local optimizer is set to $0.01/N$. An initial step size that is small and decreasing with N is central to the robust convergence of a local optimizer, due to QAOA parameters having different scales for different N. We do not find it necessary for obtaining high-quality parameters to perform objective function rescaling of the type explored in Refs. (14 and 85), though we expect that it may reduce the number of iterations required by the local optimizer to converge.

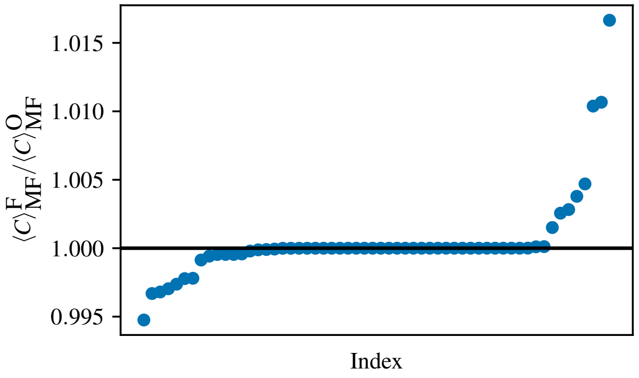

FIG. S6. **FOURIER heuristic finds good parameters** The ratio between the expected merit factor of QAOA with parameters optimized by directly running local optimization from many initial points ($\langle C\rangle^{\rm O}_{\rm MF}$) and with parameters optimized using the FOURIER$[\infty, 0]$ scheme ($\langle C\rangle^{\rm F}_{\rm MF}$) for $N > 12$. We observe that the difference in the quality of the parameters obtained by the two optimization schemes is small.

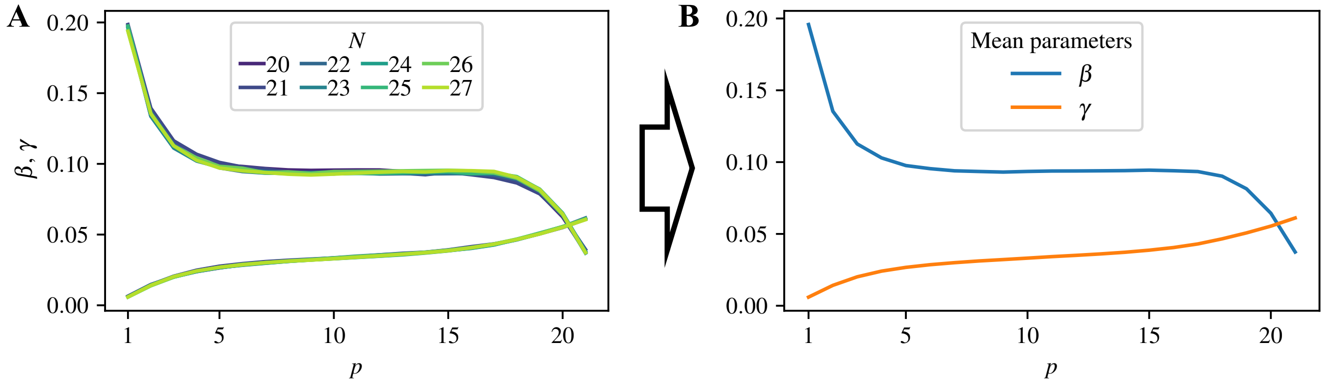

FIG. S7. **Visualization of how the fixed parameters are obtained.** Visualization of how the fixed parameters with respect to $\langle C\rangle_{\rm MF}$ are obtained. Visualization for $p^{\rm opt}$ is presented in the main text. **A,** Optimized QAOA parameters $\boldsymbol{\beta}$ (top line) and $\boldsymbol{\gamma}$ (bottom line) for $p = 21$. $\boldsymbol{\gamma}$ is scaled by $N/24$, with the constant factor of $1/24$ added for figure readability. **B,** Fixed parameters obtained by taking the arithmetic mean over the optimized parameters.

C. Evidence of the success of the FOURIER reparameterization heuristic

To evaluate the success of the FOURIER parameter optimization heuristic, we compare the quality of optimized parameters it finds with the quality of the parameters obtained by running a local optimizer with $100p$ seeds from initial points sampled uniformly from $\boldsymbol{\beta}^{\rm init} \in [0.1, 0.2]^p, \boldsymbol{\gamma}^{\rm init} \in [0, 0.85/N]^p$.

We find that the very expensive direct optimization performs very similarly to one local optimization run used in the FOURIER scheme, as shown in Figure S6. Specifically, the mean difference between the two schemes is $< 0.05\%$, and in the worst case of the ones considered, FOURIER gives parameters that are only $< 0.5\%$ worse. Therefore below, we simply consider parameters optimized using the FOURIER$[\infty, 0]$ scheme.

D. Procedure for obtaining the fixed parameters

The procedure we follow for obtaining the fixed parameters is described in the main text. We reiterate it here for completeness. We optimize QAOA parameters for smaller values of N for which the simulation is relatively inexpensive

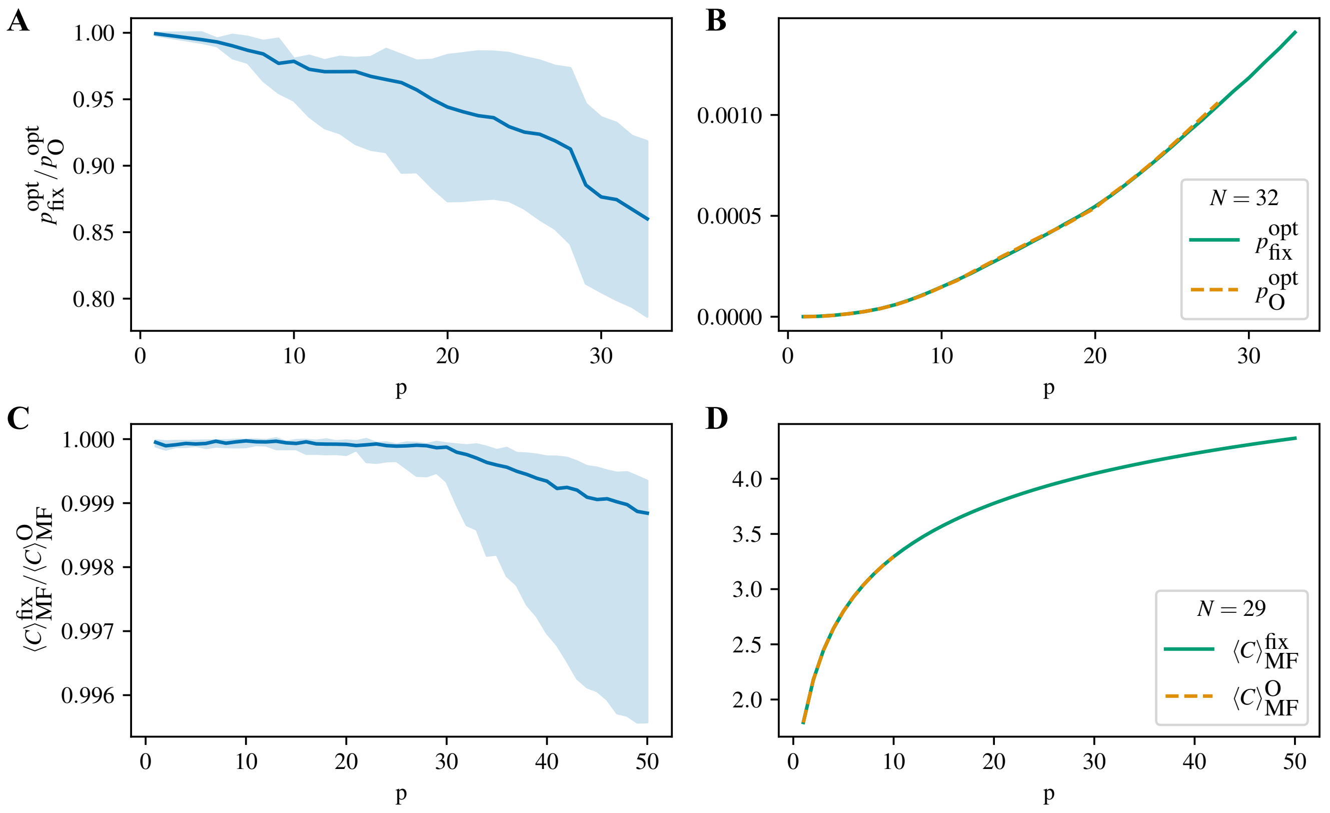

FIG. S8. **QAOA with fixed parameters performs similarly to QAOA with directly optimized parameters** Comparison of QAOA performance with fixed ($\langle C\rangle^{\rm fix}_{\rm MF}$, $p^{\rm opt}_{\rm fix}$) and optimized ($\langle C\rangle^{\rm O}_{\rm MF}$, $p^{\rm opt}_{\rm O}$) parameters. **A,C,** Shaded area shows 95% confidence interval. **B,D,** Despite the relative differences between performance with optimized and transferred parameters growing with p, we observe that for all cases considered, QAOA performance improves monotonically as expected. Since the absolute differences are small, the performance with fixed and directly optimized parameters is visually indistinguishable.

and set the fixed parameters to be the mean over the optimized parameters:

$$\boldsymbol{\beta}^{\rm Fixed} = \frac{1}{M} \sum_{N\in\{N_1,\dots,N_M\}} \boldsymbol{\beta}^*_N, \tag{S3}$$

$$\boldsymbol{\gamma}^{\rm Fixed} = \frac{1}{M} \sum_{N\in\{N_1,\dots,N_M\}} N\boldsymbol{\gamma}^*_N, \tag{S4}$$

where $\boldsymbol{\beta}^*_N$, $\boldsymbol{\gamma}^*_N$ are the QAOA parameters optimized for N. The fixed parameters used in QAOA applied to a LABS instance of size N are then given by $\boldsymbol{\beta}^{\rm Fixed}$, $\frac{\boldsymbol{\gamma}^{\rm Fixed}}{N}$. This process is visualized for parameters optimized with respect to $\langle C\rangle_{\rm MF}$ in Figure S7. We use optimized parameters for $20 \le N \le 27$ when computing parameters for $\langle C\rangle_{\rm MF}$ and $24 \le N \le 31$ for $p^{\rm opt}$.

E. Evidence of the success of the fixed parameter scheme

To evaluate the quality of fixed parameters, we compare the QAOA performance with fixed parameters and with directly optimized parameters. Figure S8 presents the comparison. We observe that the two are very close for small p, with the ratio between the two growing for higher p. Specifically, for parameters optimized with respect to the expected merit factor $\langle C\rangle_{\rm MF}$, the median difference in $\langle C\rangle_{\rm MF}$ between QAOA evaluated with fixed and directly optimized parameters is less than 0.01% for $p = 50$, with the difference even lower for smaller p (S8A). For parameters optimized with respect to $p^{\rm opt}$, the difference in $p^{\rm opt}$ is larger and growing with p (S8C). Note that due to the exponentially small value of $p^{\rm opt}$, small absolute differences (including due to precision limitations) translate into large relative

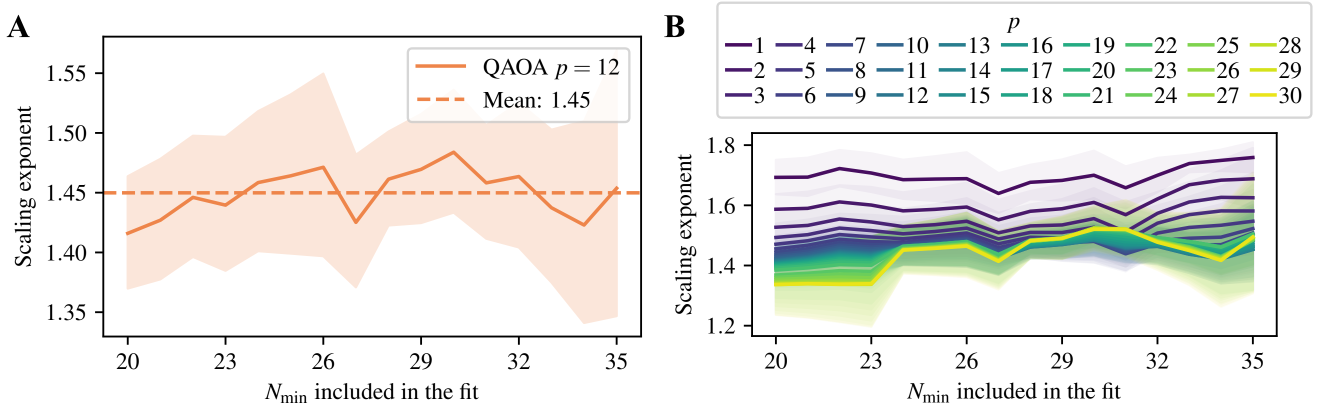

FIG. S9. **Robustness of QAOA scaling** The scaling exponent for QAOA TTS for varying choices of N_{min} included in the range of values of N for fit at **A,** $p = 12$ and **B,** for varying p. If smaller N_{min} are included, the scaling exponent continues to improve with p, with the quality of fit decaying with p (see Main text and Fig. S10). For sufficiently high N_{min}, the exponent does not improve beyond $p \approx 10$. At $p = 12$, the scaling exponent is not sensitive to the choice of N_{min} (**A**).

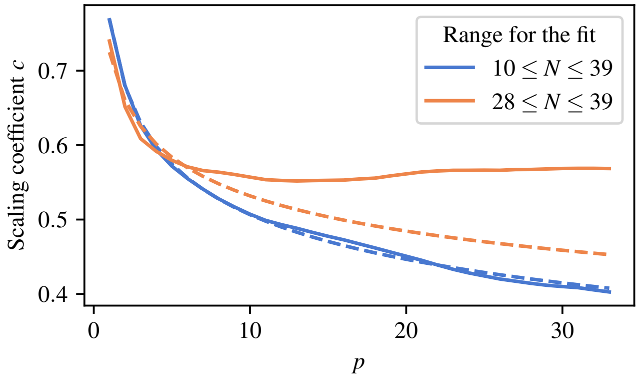

FIG. S10. **QAOA scaling does not follow power law** The scaling coefficient c in TTS $= \Theta(2^{cN})$ as a function of p. The blue line is included for illustration as including $N < 28$ leads to low quality of the fit. When the quality of the fit is high ($28 \leq N \leq 38$), the scaling coefficient does not follow a power law.

differences. Nonetheless, we observe good performance with fixed parameters at high N, as visualized for $N = 32$ in Figure S8D. QAOA performance with fixed parameters with respect to both figures of merit monotonically improves with p for all values of p considered. As the performance gap between fixed and optimized parameters grows with p, further improvements to fixed parameters are likely to yield even better scaling of QAOA TTS.

F. Scaling coefficient of QAOA TTS is not sensitive to the choice of N_{min}

In the Main text we motivate the choice of the cutoff N_{min} for the range of Ns to be included in the fit by examining the stability of the quality of fit with varying p. We obtain $N_{\text{min}} = 28$ as the minimum value required to maintain a stable fit, and estimate QAOA scaling at 1.46^N at $p = 12$. We now provide evidence that this value is not sensitive to the choice of N_{min}.

Fig. S9 shows the scaling exponent for QAOA TTS for varying choices of the cutoff N_{min}. Taking the average over exponents obtained when performing a fit with $20 \leq N_{\text{min}} \leq 35$ gives the estimated scaling of 1.45^N (Fig. S9a), which is slightly better than the one reported in the main text. As shown in Fig. S9b, for sufficiently large p and small N_{min} the exponent changes as N_{min} is increased, indicating that a larger regime of N must be explored to obtain a stable linear scaling.

G. Scaling coefficient of QAOA TTS does not follow power law

One of the important findings of this work is that for LABS problem increasing QAOA depth p beyond a certain small constant does not lead to better scaling. This puts the findings of this work in contrast to those of Refs. (13, 86, and 87). We observe that unlike in Ref. (13), the scaling coefficient c in $\mathrm{TTS} = \Theta(2^{cN})$ does not follow a power law. This is shown in Figure S10. For the coefficient c to follow a power law, it must depend on p as $c_1 \times p^{c_2}$ for some constants c_1, c_2. When the cutoff is chosen to ensure good fit ($N = 28$), we see clear deviation from a power law. We note that if we include smaller values of N in the fit and ignore the low quality of the fit, we observe a clear power law scaling of $c \sim 0.77 \times p^{-0.18}$.

H. Comparison of performance between QAOA and amplitude amplification

In this work we propose a strategy for using QAOA as a building block for algorithmic speedups by combining it with amplitude amplification (AA) or, more specifically, generalized minimum-finding as described in Appendix C of Ref. (32). Specifically, we propose running the quantum minimum-finding algorithm with constant-depth QAOA as a subroutine. If the QAOA circuit prepares the state with p^{opt} overlap with the ground state of the LABS Hamiltonian, the minimum-finding algorithm would need apply the QAOA unitary and an oracle for computing the LABS cost function $O\left(1/\sqrt{p^{\mathrm{opt}}}\right)$ times to obtain a constant probability of measuring a bitstring corresponding to the optimal solution.

We note that our numerical results suggest that increasing the QAOA depth is always beneficial as compared to doing a smaller number of QAOA steps and increasing the number of amplitude amplification steps. This is visualized in Figure S11. As this figure shows, for sufficiently high p the gain from a step of QAOA is very close to the gain from amplitude amplification, which provides an upper bound on the expected gain from one step of minimum-finding. At the same time one step of QAOA is much simpler to implement as it only requires the phase oracle and a series of one-qubit gates (mixer). We note that we do not provide exact gains from a step of the minimum-finding algorithm as they are non-trivial to estimate. However, asymptotically it is equivalent to amplitude amplification with an oracle that marks optimal solutions.

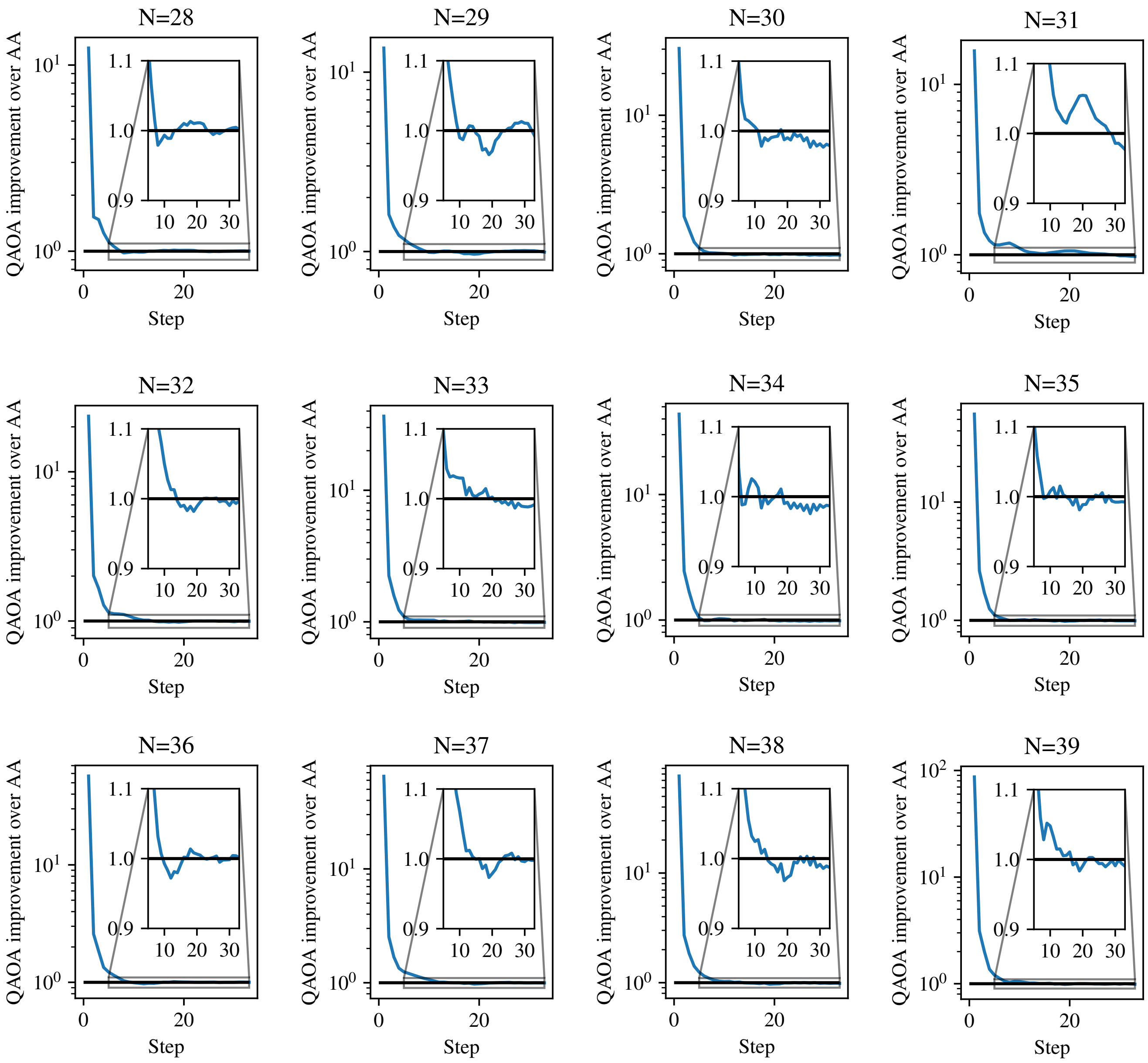

FIG. S11. **Comparison of QAOA and amplitude amplification** The ratio between the gains in the probability of measuring the optimal solution from one step of QAOA and amplitude amplification (AA). QAOA provides orders of magnitude larger improvements for the first few steps, and then behaves approximately like amplitude amplification.

I. Proof of Theorem 1

We now present the proof of Theorem 1 of the main text. Our technique is based on the generalized minimum-finding procedure outlined in Lemma 48 of Ref. (32). For reader's convenience and completeness, we begin by restating our algorithm and the Theorem.

Lemma 1 (Exponential Quantum Search, Ref. (44))**.** *Let $|\psi\rangle = U|0\rangle^{\otimes N}$ be a quantum state in a 2^N-dimensional Hilbert space with computational basis elements indexed by N-bit bitstrings, and $m\colon \{0,1\}^N \to \{0,1\}$ be a marking function such that $\sum_{\{x|m(x)=1\}} |\langle\psi|x\rangle|^2 \geq p$. There exists a quantum algorithm* **EQSearch**(U, m, δ) *that outputs an element x^* such that $m(x^*) = 1$ with probability at least $1-\delta$ using $O\left(\frac{1}{\sqrt{p}}\log\left(\frac{1}{\delta}\right)\right)$ applications of U and m.*

Algorithm 1 QAOA Enhanced with Quantum Minimum-Finding

Require: Unitary U_{QAOA} acting on $\mathbb{C}^{2^N}$ such that $|\langle x^*|U_{\text{QAOA}}|0\rangle^{\otimes N}| \geq 1/\sqrt{p_{\text{opt}}}$ for unknown p_{opt}, V_{LABS} for computing $\mathcal{E}_{\text{sidelobe}}$ into a register, and $\delta \in (0,1)$, positive number $M \leq 2^N$, C is the constant corresponding to the $O(\cdot)$ in Lemma 1

Ensure: If M is greater than $1/\sqrt{p_{\text{opt}}}$, output x^* with $\geq 1-\delta$ probability using $O(\log(1/\delta)M)$ calls to U_{QAOA} and V_{LABS} (and their inverses).

x_{res} is set to an empty list.
for $i \leftarrow 1$ **to** $\lceil \log(1/\delta) \rceil$ **do**
  $t \leftarrow 0$; $s_0 \leftarrow \infty$
  while number of calls to U_{QAOA} & V_{LABS} is $< 3CMN$ **do**
    $t \leftarrow t+1$
    Define $m_t\colon \{0,1\}^N \to \{0,1\}$ such that $m_t(x) = 1$ if and only if $\mathcal{E}_{\text{sidelobe}}(x) < s_{t-1}$. Note that m_t can be coherently evaluated using one query to V_{LABS}.
    Set $s_t = \textbf{EQSearch}(U_{\text{QAOA}}, m_t, 1/(6 \cdot 2^N))$.
  end while
  Append s_t to x_{res}.
end for
Output minimum of x_{res}.

Theorem 1. *Suppose a constant-depth QAOA circuit U_{QAOA} prepares a state $|\psi\rangle = U_{QAOA}|0\rangle^{\otimes N}$ with $N \geq 3$, such that we have $|\langle x^*|\psi\rangle| \geq 1/\sqrt{p_{opt}}$, where $|x^*\rangle$ encodes an optimal solution to the N-bit LABS problem in a computational basis state, and we assume that $p_{opt} \geq 1/N$. Then, running Algorithm 1 with parameters $M \geq 1/\sqrt{p_{opt}}$ and failure probability δ, runs with a gate complexity of $O(poly(N)\log(1/\delta)M)$ and finds x^* with probability at least $1-\delta$.*

Proof. We will first analyze the randomized Algorithm 1 as a *Las Vegas* algorithm, i.e. we assume that the internal **while** loop is infinite. In the upcoming analysis we will assume that every call to **EQSearch** in the internal loop behaves as intended. Note that we choose the failure probability of each such call to be $1/(6 \cdot 2^N)$. The total number of calls cannot be more than 2^N and so by the union bound, every call succeeds except with probability at most $1/6$.

The algorithm generates a monotonically decreasing sequence of samples, uses **EQSearch** in each iteration to search for a sample that is strictly lesser than the previous one. Since the sequence of samples is strictly decreasing and there are N possible distinct samples, the algorithm eventually returns the minimum. Since correctness is eventually guaranteed, we can simply bound the expected number of iterations before the sequence finds the minimum. If this expected number is m, we can run the internal loop $2m$ times to ensure that we find the minimum with probability at least $1/2$. Consequently, $2m \log\left(\frac{1}{\delta}\right)$ iterations suffice to ensure that we find the minimum with probability at least $1-\delta$. It remains to show that the expected number of iterations before the minimum is found is at most $O\left(\frac{1}{\sqrt{p_{\text{opt}}}}\right)$.

For this argument, we define the following quantities. $(x_1 = x^*, x_2, \ldots, x_n)$ is the list of bit-strings sorted in ascending order of the value of $\mathcal{E}_{\text{sidelobe}}$, and define $P(\xi(X))$ to be the probability of event $\xi(X)$ when X is a bitstring sampled by measuring $|\psi\rangle$ in the computational basis. Suppose in some iteration t, the sample returned by **EQSearch** is s_t. In the next iteration, **EQSearch** searches for an element with sidelobe energy less than s_t. The central observation is the following: for any x_k where $k \in [N]$, the probability that some $s_t = x_k$ given that t is the first iteration where x_k appears in the list of obtained samples is given by $P(X = x_k)/P(X \leq x_k)$. To see this, we observe as in Ref. (32) that:

$$
\begin{aligned}
\Pr(s_t = x_k | s_t \leq x_k \wedge s_{t-1} > x_k) &= \frac{\Pr(s_t = x_k)}{\Pr(s_t \leq x_k \wedge s_{t-1} > x_k)} \\
&= \sum_{x_l > x_k} \frac{\Pr(s_t = x_k \wedge s_{t-1} = x_{l-1})}{\Pr(s_t \leq x_k \wedge s_{t-1} > x_k)} \\
&= \sum_{x_l > x_k} \frac{\Pr(s_t = x_k \wedge s_{t-1} = x_{l-1}) \Pr(s_{t-1} = x_l) \Pr(s_t \leq x_k \wedge s_{t-1} = x_l)}{\Pr(s_t \leq x_k \wedge s_{t-1} > x_k) \Pr(s_{t-1} = x_l) \Pr(s_t \leq x_k \wedge s_{t-1} = x_l)} \\
&= \sum_{x_l > x_k} \frac{\Pr(s_t = x_k \wedge s_{t-1} = x_{l-1}) \Pr(s_t \leq x_k \wedge s_{t-1} = x_l)}{\Pr(s_t \leq x_k \wedge s_{t-1} > x_k) \Pr(s_t \leq x_k \wedge s_{t-1} = x_l)} \\
&= \sum_{x_l > x_k} \frac{\Pr(s_t = x_k | s_{t-1} = x_{l-1}) \Pr(s_{t-1} = x_l) \Pr(s_t \leq x_k \wedge s_{t-1} = x_l)}{\Pr(s_t \leq x_k | s_{t-1} = x_k) \Pr(s_{t-1} = x_l) \Pr(s_t \leq x_k \wedge s_{t-1} > x_l)} \\
&= \sum_{x_l > x_k} \frac{P(X = x_k) \Pr(s_{t-1} = x_l) \Pr(s_t \leq x_k \wedge s_{t-1} = x_l)}{P(X \leq x_k) \Pr(s_{t-1} = x_l) \Pr(s_t \leq x_k \wedge s_{t-1} > x_l)} \\
&= \frac{P(X = x_k)}{P(X \leq x_k)}. \qquad \text{(S5)}
\end{aligned}
$$

Notice that since a value can be sampled at most once, and the minimum is obtained within n steps, the probability that a given value x_k occurs in the list of observed samples is $P(X = x_k)/P(X \leq x_k)$.

To bound the expected number of queries before the minimum (x_1) is found by the algorithm, we associate with each bitstring $x_l \in \{x_n, x_{n\;1}, \ldots, x_2\}$ the probability that it is an obtained sample in some iteration, and the cost of performing the corresponding search for an element less than x_l. The cost of the search, for our chosen parameters is at most $\frac{C \log(6 \cdot 2^N)}{\sqrt{P(X<x_l)}} \leq \frac{2CN}{\sqrt{P(X<x_l)}}$, where the last inequality holds whenever $N \geq 3$.

The total expected number of queries before the minimum x_1 is found, is therefore given by

$$
\sum_{i=2}^{n} 2CN \cdot \frac{P(X = x_i)}{P(X \leq x_i)} \sqrt{\frac{1}{P(X < x_i)}} \leq \sum_{i=2}^{n} \frac{2CN}{P(X \leq x_i)} \sqrt{\frac{1}{P(X < x_i)}} \leq 2CN \int_{p_{\text{opt}}}^{1} r^{-3/2}\, dr \leq \frac{CN}{\sqrt{p_{\text{opt}}}}. \qquad \text{(S6)}
$$

Note that the second to last inequality follows from arguments made in Ref. (32), where they used the same inequality.

If we run the inner loop of Algorithm 1 more than 3 times the expected number of queries required to find x^*, as prescribed if $M \geq 1/\sqrt{p_{\text{opt}}}$, we fail to find x^* with probability at most 1/3 by the Markov inequality as long as no query to **EQSearch** fails. Additionally, by the earlier discussion, a query in the internal loop fails with probability at most 1/6. Therefore, by a union bound, each application of the inner loop finds x^* with probability of at least 1/2. Repeating the inner loop $\log(1/\delta)$ times ensures that x^* is found with probability at least $1 - \delta$ (if not, the inner loop has to fail to find x^* in $\log(1/\delta)$ independent trials). □

J. Details of the classical solver scaling

The scaling of the commercial branch-and-bound solvers is presented in Figure S12. For each solver, we run it with 100 random seeds for $N \leq 32$ and 10 random seeds for $N > 32$ and report the mean. The minimum N to include in the fit was chosen to maximize the quality of fit. We set the Gurobi parameters as follows: `Cuts=0`, `Heuristics=0`. For the other parameters in Gurobi and CPLEX the defaults are used. We observe that the performance of the two commercial solvers considered is within a 95% confidence interval of each other, with the TTO scaling matching that reported in Ref. (21).

We report complete results for the Memetic Tabu solver in Figure S13. The scaling is obtained by extrapolating the number of cost function evaluations made by the Memetic Tabu algorithm at different lengths. This quantity is fixed over repeated seeds for a given seed and length, unlike the execution time that may fluctuate depending on the runtime environment. The fluctuation in running time is much larger for Memetic Tabu as compared to branch-and-bound due to lower absolute value of the runtime (< 1 sec for $N = 40$) The time to evaluate the cost function on a sequence of length N scales only as N^2, which does not produce a consistent effect on runtime scaling at small lengths. The TTS scaling is therefore essentially the same as the scaling of the number of function evaluations,(30) and we choose to report the latter, more stable quantity. The seeds chosen for the runs are a contiguous block of 50

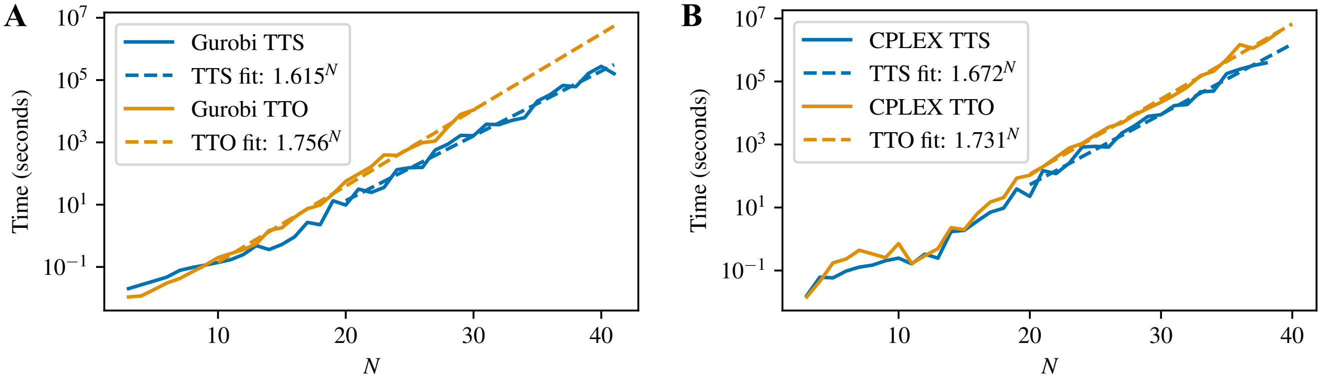

FIG. S12. **Scaling of exact solvers** Time-to-solution (TTS) and time to obtain a certificate of optimality (time-to-optimality or TTO) of **A,** Gurobi and **B,** CPLEX. For Gurobi, the 95% confidence interval (CI) for TTS is $(1.571, 1.659)$ and for TTO is $(1.721, 1.792)$. For CPLEX, the 95% CI for TTS is $(1.609, 1.737)$ and for TTO is $(1.693, 1.770)$.

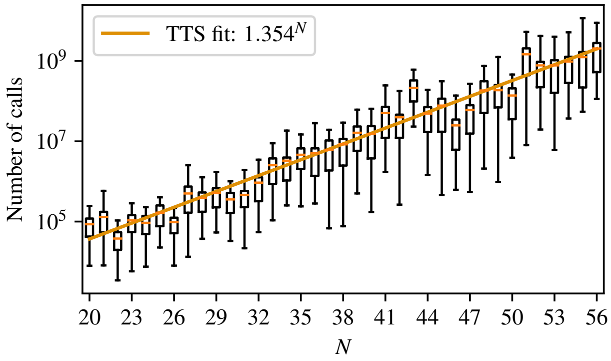

FIG. S13. **Scaling of heuristic solver**Box plots showing the run time of Memetic Tabu. The scaling fit is reported with respect to the mean. The 95% confidence interval (CI) is $(1.325, 1.383)$. The whiskers are minimum and maximum, box is showing quartiles and the horizontal line in the box is the mean.

integers chosen from a random starting point. The Memetic Tabu solver is run in single-threaded mode for all our experiments, to avoid the overestimation of cost function evaluations arising from race conditions between exploration and termination checks.

S4. EXPERIMENTS ON TRAPPED-ION SYSTEMS

A. Experimental system

The experiments in this work were performed on Quantinuum H1 and H2 platforms (26, 27). The system design is based on the QCCD architecture with multiple separate gate zones. Each gate zone is used to perform operations on an arbitrary pair of two qubits at a time, suppressing crosstalk and maintaining high fidelity. The hyperfine approximate clock states of $^{171}\mathrm{Yb}^{+}$ in the $^{2}S_{1/2}$ state are used to encode qubit information. Namely, $|0\rangle \equiv |F = 0, m_f = 0\rangle$ and $|1\rangle \equiv |F = 1, m_f = 0\rangle$. After loading, the qubits can be prepared in $|0\rangle$ via optical pumping (26, 27).

The systems have all-to-all connectivity with two-qubit gates between pairs of qubits implemented by ion transport, which brings the pairs into the same gate zone. To implement two-qubit gates, a phase-sensitive Mølmer-Sørensen (MS) gate sandwiched between single-qubit wrapper pulses is used. It in turn gives the zz gate $R_{\mathrm{zz}}(\gamma) = \exp(-i\gamma \mathrm{zz}/2)$, where the rotation angle can be precisely controlled by changing the parameters in the MS gate.(26, 27) Both the H1-1 and H2 systems used in this work have a typical average two-qubit infidelity of 2×10^{-3}, with single qubit infidelity two orders of magnitude smaller. The qubit state can be read out via state-dependent resonance fluorescence measurement.

Note that mid-circuit measurement and reset can be implemented while causing a small crosstalk error due to the stray light from the measurement and reset laser beams.

B. Circuit compilation and optimization

We now present the circuit compilation procedure for the experiments on the trapped-ion quantum processor. The H-series devices used in this work have at most five gate zones (27), i.e. at most five two-qubit gates can be executed in parallel. This implies that optimizing the circuit for full parallelism may result in diminishing returns past five parallel gates. In any case, the highest error operation on the devices is the two-qubit gate, so the primary limiting factor for achieving high-fidelity results is the two-qubit gate count. Thus, we chose to first optimize the two-qubit gate count. In addition, the cost operator is the composition of diagonal gates, and thus we are free to apply them in any order. We optimize the order to maximize the number of gate cancellations. A similar approach has been used in Ref. (88) for devices with nearest-neighbor connectivity.

We start by decomposing the four-body terms $R_{zzzz}(\gamma)$ into four CNOTs and a single $R_{zz}(\gamma)$, where $R_{zzzz}(\gamma)$ and $R_{zz}(\gamma)$ denote evolution under ZZZZ and ZZ coupling with angle γ, respectively. Note that the $R_{zz}(\gamma) = e^{-i\frac{\gamma}{2}ZZ}$ is the native gate for the Quantinuum H-series trapped-ion processors.

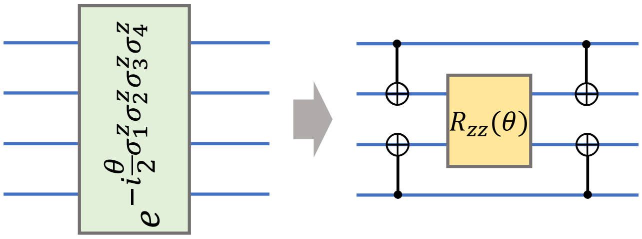

FIG. S14. **Compilation of four-body terms** Decomposition of four-body interaction terms into a two-body R_{zz} gate and four CNOT's. Figure reproduced from the main text.

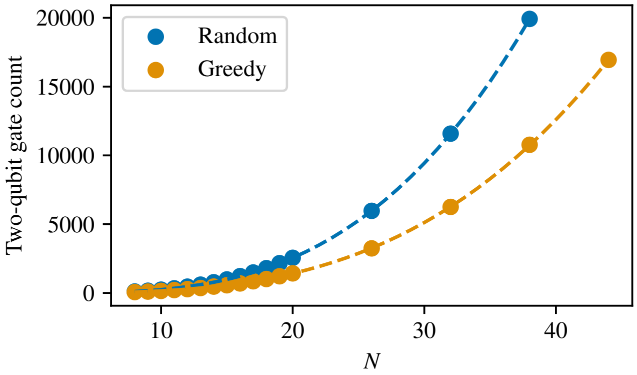

FIG. S15. **Greedy optimization reduces the gate count** Comparison of two-qubit gate count (number of $R_{zz}(\gamma)$ + $R_{zz}(\pi/2)$) of QAOA circuit at $p = 1$ with random term ordering and with the greedy optimization. The "random" line is the average over 20 random orderings of the two- and four-body terms. Both random and greedy are further optimized by tket (89), and the resulting gate count is plotted. Cubic fit line added to guide the eye.

The goal of the first step of the compilation procedure is to schedule the $R_{zzzz}(\gamma)$ gates corresponding to four-body terms in a way that greedily cancels as many CNOTs as possible (see Figure S14 for the decomposition). The second step deals with the two-body terms and attempts to schedule each $R_{zz}(\gamma)$ gate near a four-body term where one of the CNOT acts on the same qubits as the two-body term. This is to leverage two-qubit gate resynthesis implemented in tket.(89) These steps are described in detail in Algorithm 2. Then, the resulting circuit is passed to tket circuit optimizer to transpile the circuit into the H-series native two-qubit gates: $R_{zz}(\gamma)$ and $R_{zz}(\pi/2)$. The preliminary step of greedily optimizing the layout of the interactions reduced the two-qubit gate count by 1.7 times on average

compared to tket alone. The improvement in two-qubit gate count from the greedy CNOT cancellation is shown in Fig. S15. Lastly, we compared a variety of circuit optimizers and found that tket resulted in the largest gate-count reduction.

Algorithm 2 Greedy cost-operator circuit optimization

Require:

List of four-tuples of (i, j, k, l), with $i < j < k < l$, where (i, j) and (k, l), respectively correspond to the qubits of top two CNOT_{ij} and bottom two CNOT_{lk} in decomposition of R_{zzzz} presented in Figure S14. Note the indices corresponding to the control and target, respectively, are reversed for (i, j) and (k, l)

List of two-tuples (i, j), with $i < j$, indicating which qubits to apply each R_{zz} rotation to.

Ensure: Output a single list that contains all of the terms (both four- and two-body) in the order in which they should be applied, according to the greedily-optimized circuit.

circuit   empty list
for each collection of four body terms (i, j, k, l) grouped by locality $d := j - i$ $(= l - k$ for LABS$)$ **do**
  current   uniformly randomly sample (and remove) a term (i, j, k, l) from the collection of terms of locality d.
  add current to the circuit list
  tops   list initialized with tuple (i, j)
  bottoms   list initialized with tuple (k, l)
  while there are still more terms in the collection **do**
    for each term (r, s, t, v) in the collection **do**
      if $(r, s) \in$ tops or $(t, v) \in$ bottoms **then**
        Assign the term a score of $+1$
      else
        Assign the term a score of -1.
      end if
      if $\exists\, m \mid (m, r) \in$ bottoms or $\exists\, a \mid (a, t) \in$ tops **then**
        Subtract 1 from terms score. // This implies that inserting this term to the circuit would mean that there is some CNOT_{mr} or CNOT_{ta} currently in the circuit that can never be cancelled.
      end if
    end for
    current $\leftarrow$ term (a, b, c, d) in collection with highest score
    add current to the circuit list
    Add (a, b) to tops (if not already in) and (c, d) to bottoms (if not already in)
  end while
end for
for each two body term (i, j) **do**
  for each four body term (r, s, t, v) in circuit **do**
    insert (i, j) after (r, s, t, v) in circuit if $(i, j) = (r, s)$ or $(i, j) = (t, v)$ and break inner loop
  end for
  if (i, j) was not inserted into circuit **then**
    Add (i, j) to end of circuit
  end if
end for
output circuit

We note that there exists an alternative proposal (31) for reducing the cost of implementing the LABS cost operator. In this approach, the phase operator is replaced by the evolution under the Hamiltonian corresponding to

$$\mathcal{E}_{\text{sidelobe}}(\boldsymbol{s}) = \sum_{k=1}^{N-1} |\mathcal{A}_k(\boldsymbol{s})|, \tag{S7}$$

where

$$\mathcal{A}_k(\boldsymbol{s}) = \sum_{i=1}^{N-k} s_i s_{i+k}. \tag{S8}$$

This is in contrast to the approach mentioned in the main text:

$$\mathcal{E}_{\text{sidelobe}}(\boldsymbol{s}) = \sum_{k=1}^{N-1} \mathcal{A}_k^2(\boldsymbol{s}). \tag{S9}$$

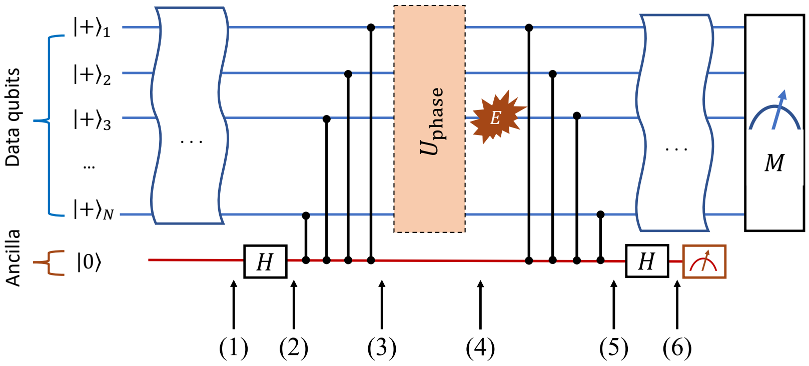

FIG. S16. **Overview of one step of the error-detection scheme.** A part of the circuit U_{phase} is "sandwiched" between two parity checks. Any error on data qubits that does not commute with the check is guaranteed to be detected.

The Hamiltonian corresponding to the absolute value of the autocorrelations has the same ground state space and energy levels as the one in Equation S9. While this reduces the asymptotic complexity for computing the energy to $\Theta(N^2)$, it requires quantum arithmetic, putting it beyond the capability of current hardware. Thus, we focus on optimizing the cost operator corresponding to Equation S9. Further gate count reductions may be possible by fixing some of the variables and applying the techniques of Ref. (90), though doing so is outside of the scope of this work.

C. Summary of the error-detection scheme

We now briefly summarize the error-detection scheme. The proof that our scheme is capable of detecting an arbitrary single-qubit error in the phase operator circuit under the assumption of noiselessly implemented parity checks is a special case of Ref. (28) (Theorem 1). We include a brief discussion of the scheme here for completeness and refer interested readers to Ref. (28) for a detailed discussion. We first consider the case of when just one parity is checked (either X or Z), and then show how the analysis generalizes to the case when both parities are checked simultaneously. Figure S16 presents the overview of the circuit. Let the state of the data qubits before the check be ρ_{init} and assume the ancilla is perfect and initialized to the pure state $|0\rangle$. The Z parity check is given by the operator $C_{\text{Z}} = \frac{1}{2}\left(\text{I} \otimes |0\rangle\langle 0| + \text{Z}^{\otimes N} \otimes |1\rangle\langle 1|\right)$. Denote the part of the phase operator sandwiched between the checks as U_{phase}. Note that U_{phase} can encompass the full phase operator or only a part of it. At (1), the state is $\rho_{(1)} = \rho_{\text{init}} \otimes |0\rangle\langle 0|$. At (2), the state is $\rho_{(2)} = \rho_{\text{init}} \otimes \frac{|0\rangle\langle 0| + |1\rangle\langle 1|}{2}$. After the first check is applied, at (3) the state becomes

$$\rho_{(3)} = \frac{1}{2}\left(\rho_{\text{init}} \otimes |0\rangle\langle 0| + \text{Z}^{\otimes N}\rho_{\text{init}}\text{Z}^{\otimes N} \otimes |1\rangle\langle 1|\right). \tag{S10}$$

If no error during the application of U_{phase} occurs, then the state at point (4) is

$$\rho_{(4)}^{\text{no error}} = \frac{1}{2}\left(U_{\text{phase}}\rho_{\text{init}}U_{\text{phase}}^{\dagger} \otimes |0\rangle\langle 0| + U_{\text{phase}}\text{Z}^{\otimes N}\rho_{\text{init}}\text{Z}^{\otimes N}U_{\text{phase}}^{\dagger} \otimes |1\rangle\langle 1|\right), \tag{S11}$$

and

$$\rho_{(5)}^{\text{no error}} = \frac{1}{2}\left(U_{\text{phase}}\rho_{\text{init}}U_{\text{phase}}^{\dagger} \otimes |0\rangle\langle 0| + \text{Z}^{\otimes N}U_{\text{phase}}\text{Z}^{\otimes N}\rho_{\text{init}}\text{Z}^{\otimes N}U_{\text{phase}}^{\dagger}\text{Z}^{\otimes N} \otimes |1\rangle\langle 1|\right) \tag{S12}$$

$$= \frac{1}{2}\left(U_{\text{phase}}\rho_{\text{init}}U_{\text{phase}}^{\dagger} \otimes |0\rangle\langle 0| + U_{\text{phase}}\rho_{\text{init}}U_{\text{phase}}^{\dagger} \otimes |1\rangle\langle 1|\right) \tag{S13}$$

$$= U_{\text{phase}}\rho_{\text{init}}U_{\text{phase}}^{\dagger} \otimes \frac{|0\rangle\langle 0| + |1\rangle\langle 1|}{2}, \tag{S14}$$

so the final state is

$$\rho_{(6)}^{\text{no error}} = U_{\text{phase}}\rho_{\text{init}}U_{\text{phase}}^{\dagger} \otimes |0\rangle\langle 0|. \tag{S15}$$

Therefore if no error occurred, measuring the ancillary qubit will always give the measurement outcome 0.

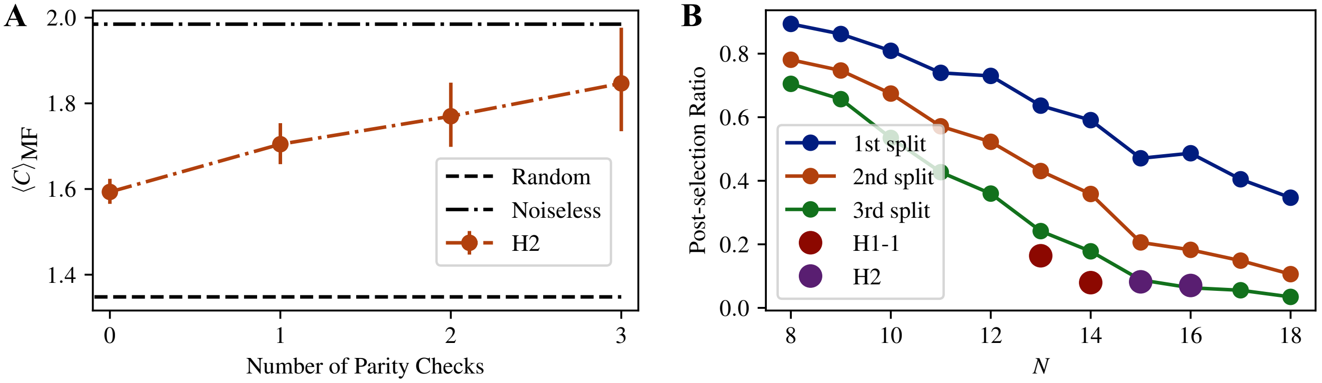

FIG. S17. **Using more parity checks gives better performance A,** Expected merit factor as a function of the number of error checks used when post-selecting the data qubit results for the $N = 15$ experiment on the H2 hardware system with 5000 repetitions. Here $m = 3$ is the number of Z- and X-parity checks. The error bars become larger as we discard more shots due to more errors being detected with more parity checks. The first data point corresponds to the case when we keep all the results from measurement on the data qubits and do not do post-selection. **B,** Post-selection ratio when splitting the phase operator into three parts and performing parity syndrome measurement at the end of each part. The curves show the simulation results using realistic parameters of the Quantinuum H2 trapped-ion device. The measured results on both H1 and H2 hardware with three checks are in good agreement with simulations.

If a single-qubit Pauli error E occurred during the execution of U_{phase}, the state at (4) becomes

$$\rho_{(4)} = \frac{1}{2}\left(EU_{\mathrm{phase}}\rho_{\mathrm{init}}U_{\mathrm{phase}}^{\dagger}E^{\dagger} \otimes |0\rangle\langle 0| + EU_{\mathrm{phase}}\mathrm{Z}^{\otimes N}\rho_{\mathrm{init}}\mathrm{Z}^{\otimes N}U_{\mathrm{phase}}^{\dagger}E^{\dagger} \otimes |1\rangle\langle 1|\right), \tag{S16}$$

and

$$\rho_{(5)} = \frac{1}{2}\left(EU_{\mathrm{phase}}\rho_{\mathrm{init}}U_{\mathrm{phase}}^{\dagger}E^{\dagger} \otimes |0\rangle\langle 0| + \mathrm{Z}^{\otimes N}EU_{\mathrm{phase}}\mathrm{Z}^{\otimes N}\rho_{\mathrm{init}}\mathrm{Z}^{\otimes N}U_{\mathrm{phase}}^{\dagger}E^{\dagger}\mathrm{Z}^{\otimes N} \otimes |1\rangle\langle 1|\right). \tag{S17}$$

If E is a Pauli, it can either commute or anti-commute with the check $\mathrm{Z}^{\otimes N}$. If it anti-commutes, then

$$\rho_{(6)} = \frac{1}{2}\left(EU_{\mathrm{phase}}\rho_{\mathrm{init}}U_{\mathrm{phase}}^{\dagger}E^{\dagger} \otimes |0\rangle\langle 0| - E\mathrm{Z}^{\otimes N}U_{\mathrm{phase}}\mathrm{Z}^{\otimes N}\rho_{\mathrm{init}}\mathrm{Z}^{\otimes N}U_{\mathrm{phase}}^{\dagger}\mathrm{Z}^{\otimes N}E^{\dagger} \otimes |1\rangle\langle 1|\right) \tag{S18}$$

$$= EU_{\mathrm{phase}}\rho_{\mathrm{init}}U_{\mathrm{phase}}^{\dagger}E^{\dagger} \otimes \frac{|0\rangle\langle 0| - |1\rangle\langle 1|}{2}, \tag{S19}$$

and the final state

$$\rho_{(6)} = U_{\mathrm{phase}}\rho_{\mathrm{init}}U_{\mathrm{phase}}^{\dagger} \otimes |1\rangle\langle 1|. \tag{S20}$$

Then measuring the ancillary qubit will return 1, and the error will be detected. A Pauli error that commutes with the check, however, will go undetected.

When both $\mathrm{X}^{\otimes N}$ and $\mathrm{Z}^{\otimes N}$ parities are checked, no odd-weight Pauli commutes with both checks. Therefore, any odd-weight Pauli error will be detected by the implemented scheme. If the checks are noiseless, increasing the frequency of checks by reducing the size of the circuit U_{phase} between the checks can only improve the final fidelity. However, in practice the checks are noisy, introducing a trade-off between the increase in the errors detected and the errors introduced by the checks themselves.

D. Performance of the error-detection scheme

In this section, we provide details on the implementation of the proposed error-detection scheme. For a circuit with m checks, we separate the phase operator into m parts, where each part has approximately the same number of two-qubit gates. We observe that increasing the frequency of the parity checks up to $m = 3$ improves the QAOA performance. Fig. S17A shows improvements in expected merit factor after post-selection. More measurements

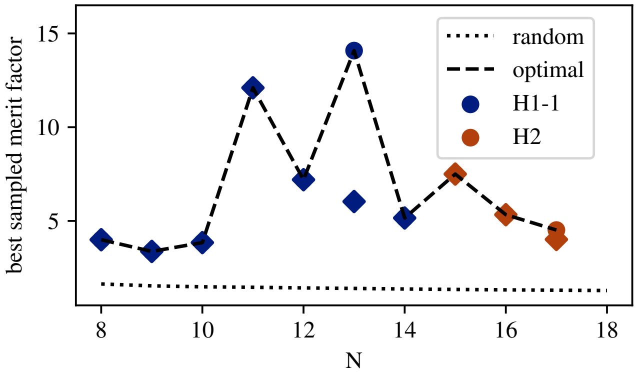

FIG. S18. **Post-selection keeps the best solution** Experimentally sampled bitstring that has the highest merit factor for different N. The number of shots taken is 2000 for $N = 8, 9$ and 5000 for other N. Circles (diamonds) are the data without (with) post-selection. They have exact same values except for $N = 13$ and $N = 17$. The best sampled bitstrings before post-selection have the same merit factor as true optimal bitstrings for all instances studied here.

increases the ratio of samples with detected errors, increasing the overhead of the error-detection scheme in terms of the number of repetitions. Fig. S17B shows how this overhead increases with N by plotting the decay of the ratio of samples with no error detected to all samples ("post-selection ratio"). This ratio drops to below 10% at $N \geq 15$ and $m = 3$. To trade off the number of repetitions required against the fidelity of the final result, we set $m = 3$ in our experiments. We further note that in our experiments, the post-selection typically keeps the bitstring with the highest merit factor sampled from experiments, as shown in Fig. S18. Note that in a practical optimization setting, the best of all bitstrings corresponding to valid solutions would be chosen as the output.

An important benefit of our error-detection scheme is reduced time to a high-quality sample, i.e. a sample with no errors detected. The time improvement comes from the ability to stop the execution when a mid-circuit measurement detects an error. This capability is particularly relevant to trapped-ion systems with relatively low clock speeds and very long coherence times enabling such classical feedback. Although available hardware supports this feature, we do not implement the early stopping in our hardware experiments. The time savings provided below are estimates.

We denote the probability that no detectable error occurs during part i as p_i. For the case without any mid-circuit syndrome measurement, the average time needed to reach a measurement result with a high merit factor, i.e., no parity error detected for all the check measurements, is given by

$$\bar{t}_1 = t_0 / (\prod_i^m p_i), \tag{S21}$$

where t_0 is the total circuit time. With mid-circuit check and feed-forward discard of the remaining circuit conditioned on the check result, the average time to get a bitstring result for which the merit factor has a high value reads as

$$\bar{t}_2 = t_0 / (\prod_i^m p_i) \times (\sum_i^{m-1} (\prod_{j=0}^{i-1} p_j (1 - p_i) \frac{i}{m}) + \prod_k^{m-1} p_k), \tag{S22}$$

with $p_0 = 1$. Here we neglect the gate time between data qubits and ancillary qubits. The comparison between the two is shown in Fig. S19, indicating that our error check method would reduce the time to get a bitstring with a high merit factor.

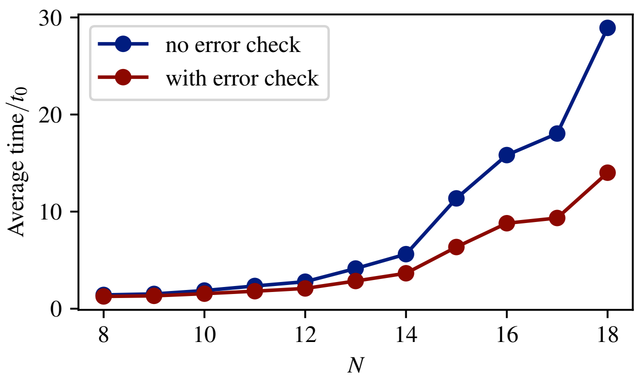

FIG. S19. **Post-selection reduces time to obtain a good bitstring** Simulation of average time (normalized by t_0) to a bitstring without detectable parity check errors. The number of splits m is set to 3. The simulations are performed using realistic parameters of the Quantinuum H2 trapped-ion device.

REFERENCES

1. C. Dürr, P. Høyer, A quantum algorithm for finding the minimum. arXiv:quant-ph/9607014 (1996).
2. A. Montanaro, Quantum-walk speedup of backtracking algorithms. *Theory Comput.* **14**, 1–24 (2018).
3. A. Montanaro, Quantum speedup of branch-and-bound algorithms. *Phys. Rev. Res.* **2**, 013056 (2020).
4. S. Chakrabarti, P. Minssen, R. Yalovetzky, M. Pistoia, Universal quantum speedup for branch-and-bound, branch-and-cut, and tree-search algorithms. arXiv:2210.03210 [quant-ph] (2022).
5. R. D. Somma, S. Boixo, H. Barnum, E. Knill. Quantum simulations of classical annealing processes. *Phys. Rev. Lett.* **101**, 130504 (2008).
6. P. Wocjan, A. Abeyesinghe, Speedup via quantum sampling. *Phys. Rev. A* **78**, 042336 (2008).
7. M. B. Hastings. A short path quantum algorithm for exact optimization. *Quantum* **2**, 78 (2018).
8. A. M. Dalzell, N. Pancotti, E. T. Campbell, F. G.S.L. Brandão, Mind the gap: Achieving a super-grover quantum speedup by jumping to the end, in *Proceedings of the ACM Symposium on Theory of Computing* (2023), pp. 1131–1144.
9. T. Hogg, D. Portnov, Quantum optimization. *Inform. Sci.* **128**, 181–197 (2000).
10. E. Farhi, J. Goldstone, S. Gutmann, A quantum approximate optimization algorithm. arXiv:1411.4028 [quant-ph] (2014).
11. L. Zhou, S.-T. Wang, S. Choi, H. Pichler, M. D. Lukin, Quantum approximate optimization algorithm: Performance, mechanism, and implementation on near-term devices. *Phys. Rev. X* **10**, 021067 (2020).
12. J. Basso, E. Farhi, K. Marwaha, B. Villalonga, L. Zhou, The quantum approximate optimization algorithm at high depth for MaxCut on large-girth regular graphs and the Sherrington–Kirkpatrick model, in *Proceedings of the Conference on the Theory of Quantum Computation, Communication and Cryptography* (2022), pp. 7:1–7:21.
13. S. Boulebnane, A. Montanaro, Solving Boolean satisfiability problems with the quantum approximate optimization algorithm. arXiv:2208.06909 [quant-ph] (2022).
14. S. H. Sureshbabu, D. Herman, R. Shaydulin, J. Basso, S. Chakrabarti, Y. Sun, M. Pistoia, Parameter setting in quantum approximate optimization of weighted problems. arXiv:2305.15201 [quant-ph] (2023).
15. A. Boehmer, Binary pulse compression codes. *IEEE Trans. Inform. Theory* **13**, 156–167 (1967).
16. M. Schroeder, Synthesis of low-peak-factor signals and binary sequences with low autocorrelation (Corresp.). *IEEE Trans. Inform. Theory* **16**, 85–89 (1970).

17. J. Bernasconi, Low autocorrelation binary sequences : Statistical mechanics and configuration space analysis. *J. Phys. France* **48**, 559–567 (1987).
18. S. Mertens, C. Bessenrodt, On the ground states of the Bernasconi model. *J. Phys. A Math. Gen.* **31**, 3731–3749 (1998).
19. M. Golay, Sieves for low autocorrelation binary sequences. *IEEE Trans. Inform. Theory* **23**, 43–51 (1977).
20. B. Bošković, F. Brglez, J. Brest, A GitHub archive for solvers and solutions of the LABS problem (2016); https://github.com/borkob/git_labs.
21. T. Packebusch, S. Mertens, Low autocorrelation binary sequences. *J. Phys. A Math. Theor.* **49**, 165001 (2016).
22. D. Lykov, R. Shaydulin, Y. Sun, Y. Alexeev, M. Pistoia. Fast simulation of high-depth QAOA circuits, in *Proceedings of the SC ’23 Workshops of The International Conference on High Performance Computing, Network, Storage, and Analysis, SC-W 2023* (ACM, 2023), pp. 1443–1451.
23. J. E. Gallardo, C. Cotta, A. J. Fernández, Finding low autocorrelation binary sequences with memetic algorithms. *Appl. Soft Comput.* **9**, 1252–1262 (2009).
24. J. Lemieux, B. Heim, D. Poulin, K. Svore, M. Troyer, Efficient quantum walk circuits for Metropolis–Hastings algorithm. *Quantum* **4**, 287 (2020).
25. S. Boixo, G. Ortiz, R. Somma, Fast quantum methods for optimization. *Eur. Phys. J. Spec. Top.* **224**, 35–49 (2015).
26. J. M. Pino, J. M. Dreiling, C. Figgatt, J. P. Gaebler, S. A. Moses, M. S. Allman, C. H. Baldwin, M. Foss-Feig, D. Hayes, K. Mayer, C. Ryan-Anderson, B. Neyenhuis, Demonstration of the trapped-ion quantum CCD computer architecture. *Nature* **592**, 209–213 (2021).
27. S. A. Moses, C. H. Baldwin, M. S. Allman, R. Ancona, L. Ascarrunz, C. Barnes, J. Bartolotta, B. Bjork, P. Blanchard, M. Bohn, J. G. Bohnet, N. C. Brown, N. Q. Burdick, W. C. Burton, S. L. Campbell, J. P. Campora III, C. Carron, J. Chambers, J. W. Chan, Y. H. Chen, A. Chernoguzov, E. Chertkov, J. Colina, J. P. Curtis, R. Daniel, M. De Cross, D. Deen, C. Delaney, J. M. Dreiling, C. T. Ertsgaard, J. Esposito, B. Estey, M. Fabrikant, C. Figgatt, C. Foltz, M. Foss-Feig, D. Francois, J. P. Gaebler, T. M. Gatterman, C. N. Gilbreth, J. Giles, E. Glynn, A. Hall, A. M. Hankin, A. Hansen, D. Hayes, B. Higashi, I. M. Hoffman, B. Horning, J. J. Hout, R. Jacobs, J. Johansen, L. Jones, J. Karcz, T. Klein, P. Lauria, P. Lee, D. Liefer, C. Lytle, S. T. Lu, D. Lucchetti, A. Malm, M. Matheny, B. Mathewson, K. Mayer, D. B. Miller, M. Mills, B. Neyenhuis, L. Nugent, S. Olson, J. Parks, G. N.

Price, Z. Price, M. Pugh, A. Ransford, A. P. Reed, C. Roman, M. Rowe, C. Ryan-Anderson, S. Sanders, J. Sedlacek, P. Shevchuk, P. Siegfried, T. Skripka, B. Spaun, R. T. Sprenkle, R. P. Stutz, M. Swallows, R. I. Tobey, A. Tran, T. Tran, E. Vogt, C. Volin, J. Walker, A. M. Zolot, J. M. Pino, A race track trapped-ion quantum processor. arXiv:2305.03828 [quant-ph] (2023).

28. A. Gonzales, R. Shaydulin, Z. H. Saleem, M. Suchara, Quantum error mitigation by Pauli check sandwiching. *Sci. Rep.* **13**, 2122 (2023).
29. D. M. Debroy, K. R. Brown. Extended flag gadgets for low-overhead circuit verification. *Phys. Rev. A* **102**, 052409 (2020).
30. B. Bošković, F. Brglez, J. Brest, Low-autocorrelation binary sequences: On improved merit factors and runtime predictions to achieve them. *Appl. Soft Comput.* **56**, 262–285 (2017).
31. Y. R. Sanders, D. W. Berry, P. C. S. Costa, L. W. Tessler, N. Wiebe, C. Gidney, H. Neven, R. Babbush. Compilation of fault-tolerant quantum heuristics for combinatorial optimization. *PRX Quantum* **1**, 020312 (2020).
32. J. van Apeldoorn, A. Gilyén, S. Gribling, R. de Wolf, Quantum SDP-solvers: Better upper and lower bounds. *Quantum* **4**, 230 (2020).
33. M. Boyer, G. Brassard, P. Høyer, A. Tapp, Tight bounds on quantum searching. *Fortschr. Phys.* **46**, 493–505 (1998).
34. R. Shaydulin, M. Pistoia, QAOA with $N \cdot p \geq 200$. arXiv:2303.02064 [quant-ph] (2023).
35. Z. He, R. Shaydulin, S. Chakrabarti, D. Herman, C. Li, Y. Sun, M. Pistoia, Alignment between initial state and mixer improves QAOA performance for constrained optimization. arXiv:2305.03857 [quant-ph] (2023).
36. E. Pelofske, A. Bärtschi, J. Golden, S. Eidenbenz, High-round QAOA for max k-sat on trapped ion NISQ devices, in *2023 IEEE International Conference on Quantum Computing and Engineering (QCE)* (IEEE, 2023), pp. 506–517.
37. E. Pelofske, A. Bärtschi, S. Eidenbenz. Quantum Annealing Vs. QAOA: 127 qubit higher-order ising problems on NISQ computers, in *Lecture Notes in Computer Science*. (Springer Nature Switzerland, 2023), pp. 240–258.
38. P. Niroula, R. Shaydulin, R. Yalovetzky, P. Minssen, D. Herman, S. Hu, M. Pistoia, Constrained quantum optimization for extractive summarization on a trapped-ion quantum computer. *Sci. Rep.* **12**, 17171 (2022).

39. R. Shaydulin, A. Galda, Error mitigation for deep quantum optimization circuits by leveraging problem symmetries, in *2021 IEEE International Conference on Quantum Computing and Engineering (QCE)* (IEEE, 2021), pp. 291–300.
40. A. Kakkar, J. Larson, A. Galda, R. Shaydulin, Characterizing error mitigation by symmetry verification in QAOA, in *IEEE International Conference on Quantum Computing and Engineering* (IEEE, 2022).
41. S. Boulebnane, A. Montanaro, Predicting parameters for the quantum approximate optimization algorithm for max-cut from the infinite-size limit. arXiv:2110.10685 [quant-ph] (2021).
42. J. Basso, D. Gamarnik, S. Mei, L. Zhou, Performance and limitations of the qaoa at constant levels on large sparse hypergraphs and spin glass models, in *2022 IEEE 63rd Annual Symposium on Foundations of Computer Science (FOCS)* (IEEE, 2022), pp. 335–343.
43. R. Babbush, J. R. McClean, M. Newman, C. Gidney, S. Boixo, H. Neven, Focus beyond quadratic speedups for error-corrected quantum advantage. *PRX Quantum* **2**, 010103 (2021).
44. G. Brassard, P. Høyer, M. Mosca, A. Tapp, Quantum amplitude amplification and estimation. *Contemp. Math.* 53–74 (2002).
45. C. N. Self, M. Benedetti, D. Amaro, Protecting expressive circuits with a quantum error detection code. arXiv:2211.06703 [quant-ph] (2022).
46. Gurobi Optimization, www.gurobi.com.
47. IBM ILOG CPLEX. V20.1: User’s manual for cplex. International Business Machines Corporation.
48. F. Glover, M. Laguna, *Tabu Search* (Kluwer Academic Publishers, 1997).
49. F. Algazi, Unified matrix treatment of the fast Walsh–Hadamard transform. *IEEE Trans. Comput.* **C-25**, 1142–1146 (1976).
50. M. Golay, A class of finite binary sequences with alternate auto-correlation values equal to zero (Corresp.). *IEEE Trans. Inform. Theory* **18**, 449–450 (1972).
51. M. J. E. Golay, D. B. Harris, A new search for skewsymmetric binary sequences with optimal merit factors. *IEEE Trans. Inform. Theory* **36**, 1163–1166 (1990).
52. G. F. M. Beenker, T. A. C. M. Claasen, P. W. C. Hermens, Binary sequences with a maximally flat amplitude spectrum. *Philips J. Res.* **40**, 289–304 (1985).
53. J. Jedwab, D. J. Katz, K.-U. Schmidt, Advances in the merit factor problem for binary sequences. *J. Comb. Theory Ser. A* **120**, 882–906 (2013).

54. M. Golay, The merit factor of long low autocorrelation binary sequences (Corresp.). *IEEE Trans. Inform. Theory* **28**, 543–549 (1982).
55. F. F. Ferreira, J. F. Fontanari, P. F. Stadler, Landscape statistics of the low-autocorrelation binary string problem. *J. Phys. A Math. Gen.* **33**, 8635–8647 (2000).
56. M. Dimitrov, T. Baitcheva, N. Nikolov, On the generation of long binary sequences with record-breaking psl values. *IEEE Signal Process. Lett.* **27**, 1904–1908 (2020).
57. J. Brest, B. Boskovic. Low autocorrelation binary sequences: Best-known peak sidelobe level values. *IEEE Access* **9**, 67713–67723 (2021).
58. B. Boskovic, J. Brest, Two-phase optimization of binary sequences with low peak sidelobe level value. arXiv:2107.09801 [cs.AI] (2021).
59. J. Lindner, Binary sequences up to length 40 with best possible autocorrelation function. *Electron. Lett.* **11**, 507 (1975).
60. S. Mertens, Exhaustive search for low-autocorrelation binary sequences. *J. Phys. A Math. Gen.* **29**, L473–L481 (1996).
61. F. Brglez, X. Y. Li, M. F. Stallmann, B. Militzer, Reliable cost predictions for finding optimal solutions to labs problem: Evolutionary and alternative algorithms, in *Proceedings of The Fifth International Workshop on Frontiers in Evolutionary Algorithms (FEA'2003) under JCIS'2003* (2003).
62. Ivan Dotu, Pascal Van Hentenryck. A note on low autocorrelation binary sequences, in *Principles and Practice of Constraint Programming - CP 2006*, F. Benhamou, Ed. (Springer Berlin Heidelberg, 2006), pp. 685–689.
63. J. Kratica, A mixed integer quadratic programming model for the low autocorrelation binary sequence problem. *Serdica J. Comput.* **6**, 385–400 (2013).
64. S. D. Prestwich, Improved branch-and-bound for low autocorrelation binary sequences. arXiv:1305.6187 [cs.AI] (2013).
65. J. Brest, B. Boskovic, A heuristic algorithm for a low autocorrelation binary sequence problem with odd length and high merit factor. *IEEE Access* **6**, 4127–4134 (2018).
66. J, E. Gallardo, C, Cotta, A. J. Fernandez, A memetic algorithm for the low autocorrelation binary sequence problem, in *Proceedings of the 9th Annual Conference on Genetic and Evolutionary Computation, GECCO07* (ACM, 2007).

67. M. B. Hastings, Classical and quantum bounded depth approximation algorithms. arXiv:1905.07047 [quant-ph] (2019).
68. C.-N. Chou, P. J. Love, J. S. Sandhu, J. Shi, Limitations of local quantum algorithms on random max-k-xor and beyond. arXiv:2108.06049 [quant-ph] (2021).
69. A. Chen, N. Huang, K. Marwaha, Local algorithms and the failure of log-depth quantum advantage on sparse random csps. arXiv:2310.01563 [quant-ph] (2023).
70. R. Shaydulin, I. Safro, J. Larson, Multistart methods for quantum approximate optimization, in *IEEE High Performance Extreme Computing Conference* (IEEE, 2019).
71. G. E. Crooks, Performance of the quantum approximate optimization algorithm on the maximum cut problem. arXiv:1811.08419 [quant-ph] (2018).
72. M. Streif, M. Leib, Training the quantum approximate optimization algorithm without access to a quantum processing unit. *Quantum Sci. Technol.* **5**, 034008 (2020).
73. X. Lee, Y. Saito, D. Cai, N. Asai, Parameters fixing strategy for quantum approximate optimization algorithm, in *International Conference on Quantum Computing and Engineering* (IEEE, 2021).
74. S. H. Sack, M. Serbyn, Quantum annealing initialization of the quantum approximate optimization algorithm. *Quantum* **5**, 491 (2021).
75. O. Amosy, T. Danzig, E. Porat, G. Chechik, A. Makmal, Iterative-free quantum approximate optimization algorithm using neural networks. arXiv:2208.09888 [quant-ph] (2022).
76. J. Larkin, M. Jonsson, D. Justice, G. G. Guerreschi, Evaluation of QAOA based on the approximation ratio of individual samples *Quantum Sci. Technol.* **7**, 045014 (2022).
77. P. C. Lotshaw, T. S. Humble, R. Herrman, J. Ostrowski, G. Siopsis, Empirical performance bounds for quantum approximate optimization. *Quantum Inf. Process.* **20**, 403 (2021).
78. L. Li, M. Fan, M. Coram, P. Riley, S. Leichenauer, Quantum optimization with a novel Gibbs objective function and ansatz architecture search. *Phys. Rev. Res.* **2**, 023074 (2020).
79. P. K. Barkoutsos, G. Nannicini, A. Robert, I. Tavernelli, S. Woerner, Improving variational quantum optimization using CVaR. *Quantum* **4**, 256 (2020).
80. R. Shaydulin, S. Hadfield, T. Hogg, I. Safro, Classical symmetries and the quantum approximate optimization algorithm. *Quantum Inf. Process.* **20**, 359 (2021).
81. S. Bravyi, A. Kliesch, R. Koenig, E. Tang, Obstacles to variational quantum optimization from symmetry protection. *Phys. Rev. Lett.* **125**, 260505 (2020).

82. Z. Wang, S. Hadfield, Z. Jiang, E. G. Rieffel, Quantum approximate optimization algorithm for MaxCut: A fermionic view. *Phys. Rev. A* **97**, 022304 (2018).
83. S. G. Johnson, The NLopt nonlinear-optimization package (2022). http://github.com/stevengj/nlopt.
84. M. J. D. Powell, "The BOBYQA algorithm for bound constrained optimization without derivatives" (Cambridge NA Report NA2009/06, University of Cambridge, 2009).
85. R. Shaydulin, P. C. Lotshaw, J. Larson, J. Ostrowski, T. S. Humble, Parameter transfer for quantum approximate optimization of weighted MaxCut *Comput. Secur.* **4**, 1–15 (2023).
86. P. C. Lotshaw, G. Siopsis, J. Ostrowski, R. Herrman, R. Alam, S. Powers, T. S. Humble, Approximate Boltzmann distributions in quantum approximate optimization. *Phys. Rev. A* **108**, 042411 (2023).
87. V. Akshay, H. Philathong, E. Campos, D. Rabinovich, I. Zacharov, X.-M. Zhang, J. D. Biamonte. Circuit depth scaling for quantum approximate optimization. *Phys. Rev. A* **106**, 042438 (2022).
88. J. Unger, A. Messinger, B. E. Nieho, M. Fellner, W. Lechner, Low-depth circuit implementation of parity constraints for quantum optimization. arXiv:2211.11287 [quant-ph] (2022).
89. S. Sivarajah, S. Dilkes, A. Cowtan, W. Simmons, A. Edgington, R. Duncan. t|ket⟩: A retargetable compiler for NISQ devices. *Quantum Sci. Technol.* **6**, 014003 (2021).
90. R Ayanzadeh, N Alavisamani, P. Das, M. Qureshi, FrozenQubits: Boosting fidelity of QAOA by skipping hotspot nodes, in *International Conference on Architectural Support for Programming Languages and Operating Systems* (ACM, 2023), pp. 311–324.
